# PKD3 localizes to late endosomes to maintain Rab7-dependent endolysosomal homeostasis

**DOI:** 10.1016/j.isci.2025.113408

**Published:** 2025-08-20

**Authors:** Elena Gutiérrez-Galindo, Katharina Jursik, Yannick Frey, Florian Meyer, Angelika Hausser

**Affiliations:** 1 Institute of Cell Biology and Immunology, University of Stuttgart, Allmandring 31, 70569 Stuttgart, Germany; 2 Institute of Pathophysiology, Medical University of Innsbruck, Innrain 80/82, 6020 Innsbruck, Austria; 3 Stuttgart Research Center Systems Biology, University of Stuttgart, Nobelstr. 15, 70569 Stuttgart, Germany

**Keywords:** Biological sciences, Molecular biology, Developmental biology

## Abstract

Protein kinase D3 (PKD3) is an important regulator of triple-negative breast cancer (TNBC) progression by promoting invasion, proliferation, and stem cell maintenance. However, the mechanism underlying these cellular functions has remained unclear. Here, we report that endogenous PKD3 localizes to Rab7-positive vesicles in MDA-MB-231 cells cultured on stiff matrices. Notably, PKD3 depletion results in smaller Rab7-positive vesicles with reduced retromer complex recruitment, leading to enhanced cathepsin D secretion. This correlates with impaired endosomal acidification, which is associated with dysregulated Wnt signaling and a decline in stemness. Our data thus unveil a previously unrecognized role of PKD3 in regulating endolysosomal dynamics that contributes to the maintenance of the cancer stem cell population in TNBC.

## Highlights

• Endogenous PKD3 localizes to membranes of the endolysosomal compartment
• PKD3 maintains Rab7 and retromer recruitment to late endosomes
• PKD3 loss results in impaired lysosomal acidification
• PKD3 function supports Wnt signaling to promote breast cancer stemness

## Graphical abstract

**Figure:**
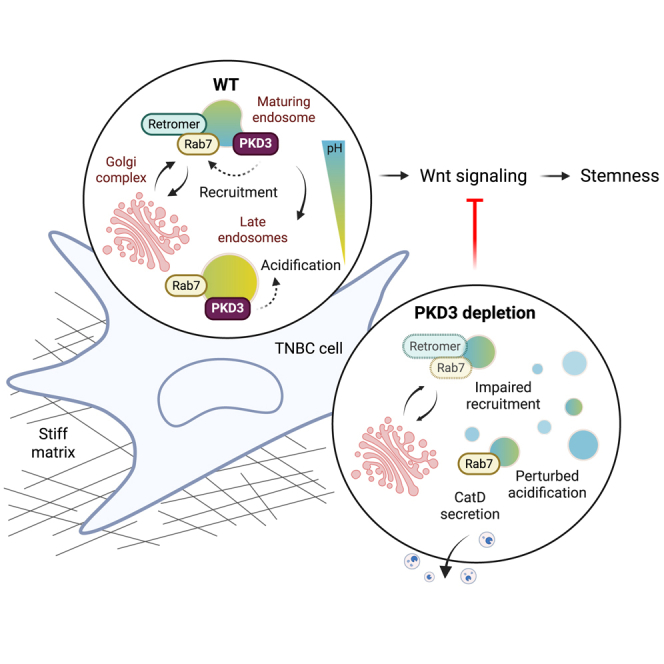

## Introduction

The serine/threonine Protein Kinase D family (PKD, comprising PKD1, PKD2, and PKD3) is involved in several cellular processes, including cell migration,^1^^,^^2^ proliferation,^3^^,^^4^^,^^5^ protein transport,^6^^,^^7^^,^^8^^,^^9^ secretion^10^^,^^11^ and EMT,^12^ and thus has a key role in tumor progression.

PKD exists as an inhibitory dimer in the cytosol and is activated when bound to endomembranes in a diacylglycerol (DAG)-dependent manner.^13^ Consistent with its localization to endomembranes, numerous studies confirm a function of the PKD family in regulating membrane trafficking (reviewed in^14^), particularly in the secretory pathway, and assign it a major function at the trans-Golgi-network (TGN).^8^^,^^9^^,^^15^ However, while it has been speculated that PKD2 and PKD3 isoforms can form homo- and heterodimers to control secretion at the level of the Golgi,^6^ little is known about the spatiotemporal regulation and localization of the different isoforms and how this affects PKD-dependent functions at the endogenous level.

In triple-negative breast cancer (TNBC), PKD2 and PKD3 are expressed, with PKD3 being the predominant isoform.^5^^,^^16^ Recently, an important role for PKD2 in regulating the pro-invasive secretome of TNBC cells has been described,^10^ while PKD3 was shown to be essential to maintain the stem cell population in TNBC cells in both *in vitro* and *in vivo*.^17^ However, the mechanism by which PKD3 controls this process remained unknown.

Here, we provide evidence that endogenous PKD3 localizes to membranes of the endolysosomal compartment to control Rab7 localization and, as a consequence, retromer complex distribution, Cathepsin D (CatD) secretion, and endolysosomal acidification. Additionally, we found that this role of PKD3 in preserving endolysosomal homeostasis and function supports stemness in TNBC via modulating the Wnt pathway.

## Results

### Active protein kinase D3 localizes to membranes of the endolysosomal compartment

**Figure 1:**
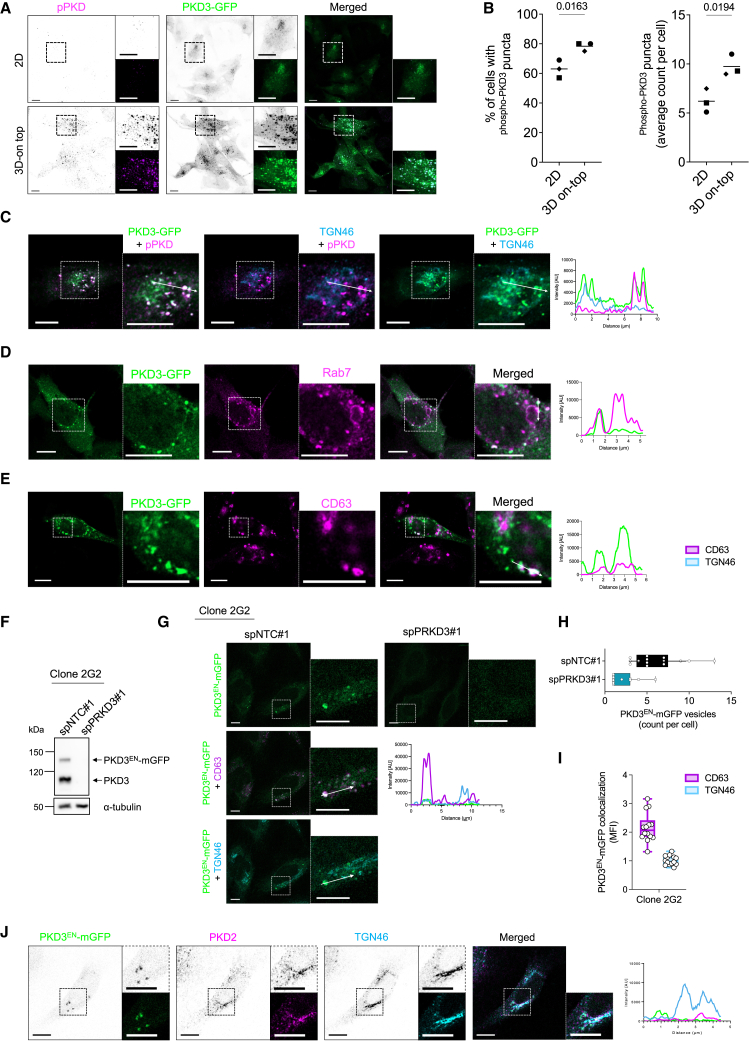
Active PKD3 localizes to membranes of the endolysosomal compartment (A) Maximum intensity projections (MIP) of MDA-MB-231_PKD3-GFP cells seeded on glass coverslips (2D) or 5 kPa PAA hydrogels (3D-on top), fixed and stained for pPKD (magenta); scale bar: 10 μm. (B) Quantification of A); *n* = 3, *N* > 35 cells per condition and experiment; statistical comparison by unpaired t-test. (C–E) MDA-MB-231_PKD3-GFP cells were seeded on 5 kPa PAA hydrogels, fixed and stained for pPKD (magenta) and TGN46 (blue) in C), Rab7 (magenta) in D) and CD63 (magenta) in E); a middle Z-section is shown; scale bar: 10 μm; the histogram represents the intensity profile in the area marked with a white arrow in the merged image. (F) Whole cell lysates of HeLa_ PKD3^EN^-mGFP (Clone 2G2) transiently transfected with spNTC#1 or spPRKD3#1 were subjected to Western blot analysis and probed for PKD3; α-tubulin was used as a loading control. (G) HeLa_PKD3^EN^-mGFP cells (Clone 2G2) transiently transfected with spNTC#1 or spPRKD3#1 were seeded on glass coverslips and fixed; control samples were stained for CD63 (magenta) and TGN46 (blue); scale bar: 10 μm; the histogram represents the intensity profile in the area marked with a white arrow. (H–I) Quantification of G). (H) The graph represents the number of PKD3^EN^-mGFP positive vesicles per cell; *N* > 10; *n* = 1; (I) Colocalization analysis of PKD3^EN^-mGFP with CD63 and TGN46, analyzed as the MFI of PKD3^EN^-mGFP on CD63-or TGN46-positive vesicles; *N* > 10; *n* = 1. (J) HeLa_ PKD3^EN^-mGFP cells (Clone 1H4) were seeded on glass coverslips, fixed, and stained for PKD2 (magenta) and TGN46 (blue); a middle Z-section is shown; scale bar: 10 μm; the histogram represents the intensity profile in the area marked with a white arrow in the merged image. See also Figures S1–S5.

To study the molecular role of PKD3 in TNBC cells, we generated MDA-MB-231 cells overexpressing PKD3-GFP in a doxycycline (Dox)-inducible manner. Surprisingly, active PKD3, visualized by the staining of the phosphorylated activation loop (S731/735, pPKD), was not detected at a perinuclear compartment but rather localized to puncta (Figure 1A). However, since this dominant punctate localization of active PKD3 was only observed in a few cells, we moved to a more physiological 3D-on top culture system that resembles the stiffening of the tumor occurring during BC progression.^18^^,^^19^^,^^20^ When cells were cultured on 5 kPa polyacrylamide (PAA) hydrogels, significantly more cells exhibited punctate localization of PKD3 compared to standard 2D culture conditions, and the average number of active PKD3 puncta per cell was also significantly higher (Figure 1B). Consequently, all further experiments with MDA-MB-231 cells were performed using the 3D-on top culture system, which appears to favor elevated PKD3 activity.

PKD is active when bound to endomembranes^21^ and, in epithelial cells, the ectopically expressed protein localizes to the TGN to control the fission of vesicles containing cargo for the plasma membrane.^8^^,^^9^^,^^15^ Breast cancer cell lines often harbor a fragmented Golgi apparatus.^22^ Hence, to investigate whether the vesicles on which PKD3-GFP was detected might originate from the TGN, we stained PKD3-GFP expressing MDA-MB-231 cells with an antibody specific for TGN46. To our surprise, active PKD3-GFP (pPKD) was frequently localized near perinuclear, TGN46 positive membranes, but did not co-localize with them (Figure 1C). We therefore tested different markers for endosomal membranes and found that a pool of active PKD3-GFP co-localized with Rab7 (Figures 1D and S1A), with CD63 (Figure 1E), and to a smaller extent with Lamp1 vesicles (Figure S1B), all well-established components of late endosomal and lysosomal membranes.^23^ In line with this, active PKD3-GFP was often found to decorate the membrane of acidic vesicles labeled with LysoTracker (Figure S1C).

To rule out that this localization is a result of the ectopic overexpression of PKD3-GFP, we used CRISPR/Cas12a assisted PCR tagging^24^ to fuse endogenous PKD3 (PKD3^EN^) with mGFP. Since our tagging approach was not successful in MDA-MB-231 cells, we employed the well-established HeLa cells that express PKD2 and PKD3, but not PKD1,^7^^,^^25^ as a model. In brief, after transfection and antibiotic selection to generate two independent HeLa_PKD3^EN^-mGFP pools, PKD3^EN^-mGFP expression was controlled by Western blot (Figure S2A), and a subsequent limited dilution of Pool 2 was performed to select positive single clones. Out of 32 clones, 7 were positive for PKD3^EN^-mGFP expression, and clones 1H4 and 2G2 were chosen for further experiments (Figure S2B). Of note, all clones only showed a partial tagging efficiency since the expression of untagged PKD3 was still detectable. The sequencing of the genomic DNA of both clones showed the correct in the frame insertion of mGFP at the C-terminus of PKD3 (Figure S3). In addition, transient transfection with a PKD3 specific siRNA (spPRKD3#1) proved specificity as PKD3^EN^-mGFP levels decreased together with untagged PKD3 for Pool 2 (Figure S2A) and both clones, 2G2 (Figures 1F) and 1H4 (Figure S4A). Confocal microscopy studies of the two clones revealed a vesicular localization of PKD3^EN^-mGFP that was absent in the knockdown condition (Figures 1G, 1H, S4B, and S4C). More precisely, quantification showed that these PKD3^EN^-mGFP vesicles co-localized with CD63, but not with the perinuclear TGN (detected with TGN46), for both 2G2 (Figure 1I) and 1H4 clones (Figure S4D). As previously described for MDA-MB-231_PKD3-GFP cells, these PKD3^EN^-mGFP positive, late endosomal membranes were also positive for Rab7 (Figure S5), confirming our findings.

The formation of homo-and heterodimers for all PKD isoforms has been reported in different studies using recombinant proteins,^6^^,^^26^^,^^27^ but whether these are formed at the endogenous level in intact cells still remains unclear. Since HeLa cells express PKD2 but not PKD1, we stained endogenous PKD2 in HeLa_PKD3^EN^-mGFP cells using a specific antibody. We found a co-localization of PKD2 with TGN46, but no evident overlap with PKD3^EN^-mGFP (Figure 1J). This suggests that the majority of endogenous PKD2 and PKD3 exist as separate entities rather than forming heterodimers under basal conditions.

Taken together, our observations indicate a previously unrecognized localization of endogenous PKD3 to membranes of the endolysosomal compartment.

### Loss of PKD3 leads to a redistribution of Rab7

To further explore the role of PKD3 at endolysosomes, we investigated possible binding partners of the kinase at endolysosomes. Two independent publications detected by proximity label-mass spectrometry an interaction of PKD3 with Rab7A (hereafter Rab7),^28^^,^^29^ which is well known to be the key regulator of early-to-late endosome maturation and motility, lysosomal biogenesis, and cargo transport^30^ and which we found decorating PKD3-GFP positive vesicles (Figures 1D and S1A).

**Figure 2:**
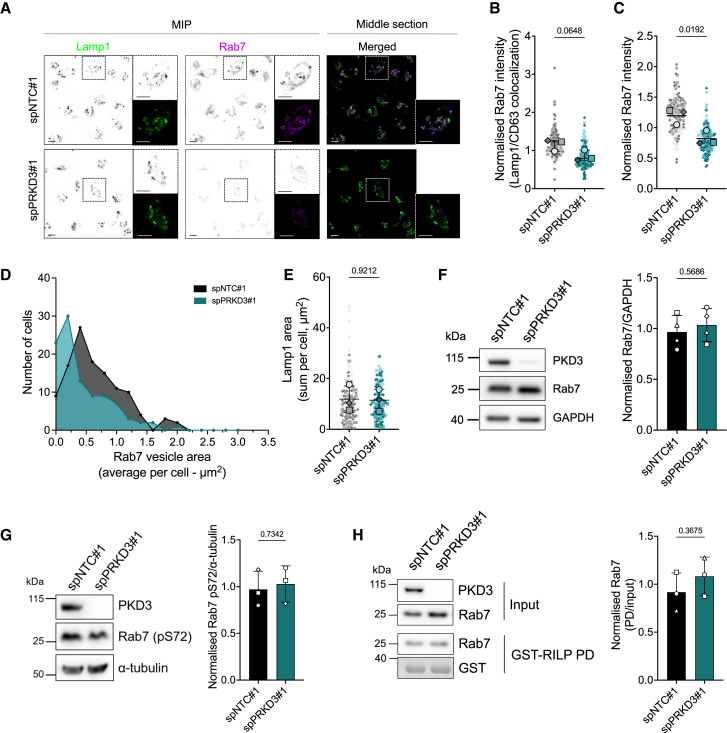
Loss of PKD3 leads to a redistribution of Rab7 (A) MDA-MB-231 cells transiently transfected with spNTC#1 or spPRKD3#1 were seeded on 5 kPa PAA hydrogels, fixed and stained as indicated: Lamp1 (green) and Rab7 (magenta); black and white images show maximum intensity projections (MIP), and colored images show a middle Z-section; scale bar: 10 μm. B–E) Quantification of A); *n* = 3; *N* > 35 cells per condition and experiment. (B) Colocalization of Rab7 with Lamp1 or CD63, analyzed as the MFI of Rab7 on Lamp1-or CD63-positive vesicles; statistical comparison by unpaired t-test. (C) Rab7 MFI of MIPs, normalized to the average MFI for each independent experiment; statistical comparison by unpaired t-test. (D) Frequency of the distribution of Rab7-positive vesicles from three independent experiments, distributed by area. (E) Area of Lamp1-or CD63-positive vesicles (sum per cell). *n* = 3; *N* > 35 cells per condition and experiment; statistical comparison by unpaired t-test. (F–G) Whole cell lysates of MDA-MB-231 cells transiently transfected with spNTC#1 or spPRKD3#1 were subjected to Western blot analysis and probed for: PKD3 and Rab7 in F) and PKD3 and Rab7 (pS72) in G); GAPDH (F) or α-tubulin (G) were used as a loading control; the blots are representative of four (F) or three (G) independent experiments, and the graphs show the quantification; statistical comparison by unpaired t-test. (H) Whole cell lysates of MDA-MB-231 cells transiently transfected with spNTC#1 or spPRKD3#1 were subjected to pull-down assays using GST-RILP and analyzed by Western blot using a Rab7 antibody. The blot is representative of three independent experiments, and the graph shows the quantification; statistical comparison by unpaired t-test.

To investigate whether PKD3 can be functionally linked to Rab7, MDA-MB-231 cells transiently transfected with a control siRNA (spNTC#1), or a PKD3-specific siRNA (spPRKD3#1) were stained for Rab7, along with the lysosomal proteins Lamp1 or CD63. In control cells, Rab7 was enriched at vesicular membranes, partly overlapping with Lamp1/CD63 (Figure 2A). The depletion of PKD3 reduced Rab7 localization to Lamp1-/CD63-positive membranes (Figure 2B) and Rab7 appeared to be more distributed throughout the cell (Figure 2A). The quantification of the Rab7 signal showed a decrease in the mean fluorescence intensity (MFI) in PKD3-depleted cells compared to control cells (Figure 2C). Additionally, the Rab7-positive vesicles were analyzed to compare size and count, demonstrating a shift from larger to smaller vesicles upon PKD3 depletion (Figure 2D), while the area of Lamp1-/CD63-positive vesicles remained unchanged (Figure 2E). Western blot analysis confirmed the equal expression of Rab7 in all conditions analyzed, supporting that the differences in fluorescence intensity are due to the redistribution of Rab7 rather than loss of the protein (Figure 2F). Notably, this redistribution was not accompanied by a detectable change in Rab7 activity, as GST-RILP pulldown assays showed no differences between control and PKD3-depleted cells (Figure 2H), ruling out a cytosolic localization of Rab7. In addition, the phosphorylation of Rab7 at serine 72 has been linked to membrane association and effector binding.^31^ However, we did not detect any significant differences in Rab7 phosphorylation between control and PKD3-depleted cells (Figure 2G). While Rab7 is predominantly localized to late endosomes and lysosomes, our findings align with previous reports indicating that Rab7 can also associate with small vesicles, the nature of which remains to be determined.^23^^,^^32^

### Loss of protein kinase D3 perturbs retromer recruitment to Rab7-positive vesicles

**Figure 3:**
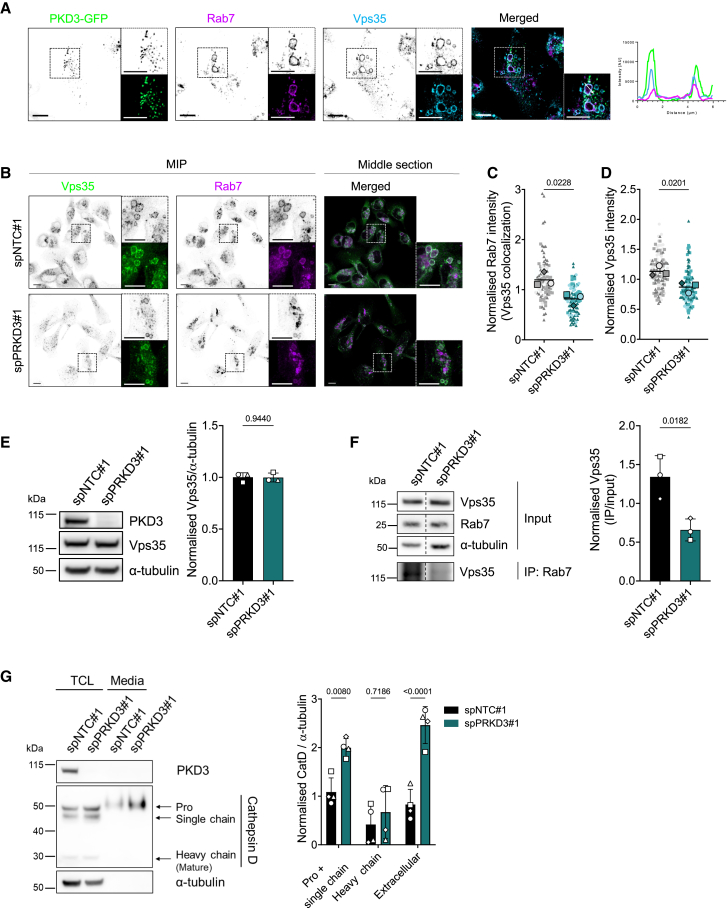
Loss of PKD3 perturbs retromer function (A) MDA-MB-231_PKD3-GFP cells were seeded on 5 kPa PAA hydrogels, fixed, and stained for Rab7 (magenta) and Vps35 (blue); a middle Z-section is shown; scale bar: 10 μm; the histogram represents the intensity profile in the area marked with a white arrow in the merged image. (B) MDA-MB-231 cells transiently transfected with spNTC#1 or spPRKD3#1 were seeded on 5 kPa PAA hydrogels, fixed and stained as indicated: Vps35 (green) and Rab7 (magenta); black and white images show MIPs, and colored images show a middle Z-section; scale bar: 10 μm. (C and D) Quantification of B). C) Colocalization of Rab7 and Vps35, analyzed as the MFI of Rab7 on Vps35-positive vesicles; *n* = 3, *N* > 15 cells per condition and experiment. D) Vps35 MFI of MIPs, normalized to the average MFI for each independent experiment; statistical comparison by unpaired t-test; *n* = 3, *N* > 30 cell per condition and experiment. (E) Whole cell lysates of MDA-MB-231 cells transiently transfected with spNTC#1 or spPRKD3#1 were subjected to Western blot analysis and probed for PKD3 and Vps35; α-tubulin was used as a loading control. The blots are representative of three independent experiments, and the graph shows the quantification; statistical comparison by unpaired t-test. (F) Whole cell lysates of MDA-MB-231 cells transiently transfected with spNTC#1 or spPRKD3#1 were subjected to co-immunoprecipitation assays using a Rab7 antibody, analyzed by Western blot and probed for Vps35 and Rab7; α-tubulin was used as a loading control. The vertical, dashed line indicates a cropped lane from the same blot. The blot is representative of three independent experiments, and the graph shows the quantification; statistical comparison by unpaired t-test. (G) Total cell lysates (TCL) and supernatant of MDA-MB-231 cells transiently transfected with spNTC#1 or spPRKD3#1 were subjected to Western blot analysis and probed for PKD3 and CatD; α-tubulin was used as a loading control; the blot is representative of four independent experiments, and the graph shows the quantification; statistical comparison by two-way ANOVA with Šídák’s multiple comparisons test.

Previous studies have demonstrated that Rab7 localization to late endosomal membranes is associated with the recruitment of the retromer complex.^32^^,^^33^ This multiprotein complex regulates the sorting of integral membrane proteins through the endolysosomal network and plays a critical role in Rab7 nucleotide cycling.^34^^,^^35^^,^^36^^,^^37^ Remarkably, we observed that PKD3-GFP decorated the membrane of enlarged Rab7-positive vesicles, where the retromer subunit Vps35 was also found (Figure 3A). Furthermore, since PKD3 expression modulated the enrichment of Rab7 at endosomal membranes and Rab7 is closely connected with the retromer complex, we hypothesized that the recruitment of the retromer complex to these membranes could follow a similar pattern. Indeed, the co-staining of Rab7 along with the retromer subunit Vps35 in MDA-MB-231 cells confirmed a reduced co-localization of both proteins when PKD3 was depleted (Figures 3B and 3C). The quantification of the Vps35 signal also showed an overall reduced recruitment of retromer to endomembranes (Figure 3D), while Vps35 protein levels remained unchanged (Figure 3E). In line with this, co-immunoprecipitation experiments showed that the amount of Vps35 interacting with Rab7 in control cells was significantly reduced in PKD3-depleted cells (Figure 3F). These findings suggest that the retromer function could be potentially modulated by endolysosomal PKD3. A prominent cargo of the retromer complex are the lysosomal cathepsins,^32^^,^^38^ whose proper trafficking and maturation along the endolysosomal pathway is Rab7-dependent.^32^ We therefore analyzed the trafficking of CatD by Western blot analysis (Figure 3G). In control cell lysates, three protein bands were detected, presenting pro-CatD (52 kDa), an active intermediate single chain CatD (48 kDa), and the mature heavy chain (34 kDa).^39^ Notably, in PKD3-depleted cells, the immature, endosome-localized forms of CatD were significantly enriched in the lysate compared to control cells, suggesting the deregulated trafficking of CatD within the endolysosomal compartment. Previous studies have demonstrated that the disrupted recruitment of retromer to endosomes impairs the trafficking of lysosomal sorting receptors, leading to the secretion of CatD.^31^ Consistent with this, we observed a significant increase in pro-CatD levels in the supernatant following PKD3 depletion (Figure 3G). Our results are in line with previous findings^5^ and propose a role for PKD3 in coordinating endolysosomal trafficking and, consequently, lysosomal function via Rab7.

### Protein kinase D3 supports endolysosomal acidification to regulate the Wnt pathway

In TNBC cells, PKD3 has been functionally linked to the maintenance of the stem cell population.^17^ Likewise, the Wnt/β-catenin signaling pathway, which is one of the major contributors in maintaining stemness^40^ is elevated in breast cancer stem cells.^41^ Lysosomal function is closely linked to stem cell maintenance in breast cancer,^42^^,^^43^^,^^44^ while Wnt signaling has been linked to an increase in the acidity of the endolysosomal compartment and lysosomal activity.^45^^,^^46^^,^^47^ These connections suggest that our findings on PKD3 localization and its role in endolysosomal function may critically influence Wnt signaling and, consequently, cancer stemness.

**Figure 4:**
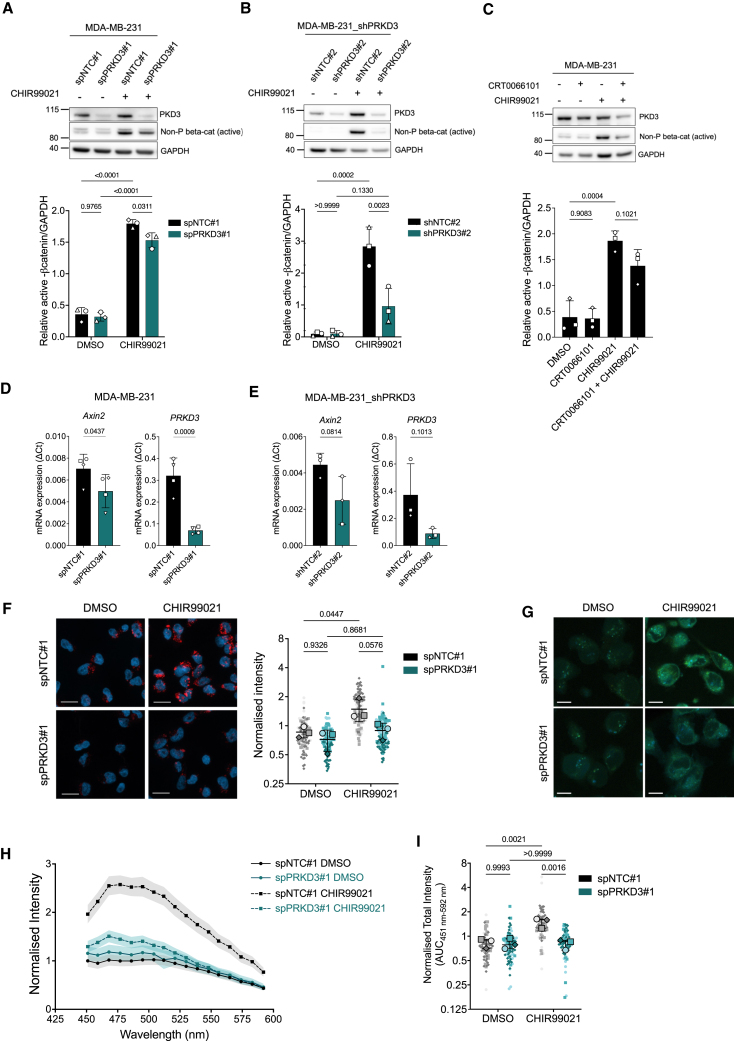
PKD3 supports lysosomal acidification to regulate the Wnt pathway (A–C) Western blot analysis of cells seeded on 5 kPa PAA hydrogels, treated as indicated: MDA-MB-231 transiently transfected with spNTC#1 or spPRKD3# (A) or stably expressing shNTC#2 or shPRKD3#2 (B) were treated with DMSO or CHIR99021 (8 nM) for 2 h; C) MDA-MB-231 cells were treated with DMSO or CRT0066101 (2.5 μM) for 1 h prior treatment with DMSO or CHIR99021 (8 nM) for 2 h; whole cell lysates were subjected to Western blot analysis and probed for PKD3 and non-phosphorylated β-catenin; GAPDH was used as a loading control; the upper panels show a representative experiment and the graphs below show the relative levels of non-phosphorylated β-catenin in three independent experiments, normalized to GAPDH levels; statistical comparison by two-way ANOVA with Šídák’s multiple comparisons test (A-B) or ordinary one-way ANOVA with Holm-Šídák’s multiple comparisons test (C). (D and E) qPCR analysis of *Axin1* and *PRKD3* in cells seeded on 5 kPa PAA hydrogels; D) MDA-MB-231 cells were transiently transfected with spNTC#1 or spPRKD3#1; *n* = 4; E) MDA-MB-231 cells stably expressed shNTC#2 or shPRKD3#2; *n* = 3); statistical comparison by t-test. (F–I) MDA-MB-231 cells transiently transfected with spNTC#1 or spPRKD3#1 were seeded on 5 kPa PAA hydrogels, treated with DMSO or CHIR99021 (8 nM) for 2 h, incubated with LysoTracker (F) or LysoSensor (G) and fixed; F) Lysotracker; scale bar: 20 μm; data in graph represents the MFI, normalized to the average MFI for each independent experiment; statistical comparison by two-way ANOVA with Šídák’s multiple comparisons test; *n* = 3, *N* > 20 cells per condition and experiment. (H) LysoSensor intensity measured in the 450–600 nm range per vesicle; scale bar: 10 μm; data in graph represent the average acidic vesicle intensity per cell, normalized to the average intensity for each independent experiment; the graph shows a representative experiment, and values are expressed in mean ±95% CI. (I) LysoSensor intensity: Area under the curve (AUC) of three independent experiments; N > 5–30 cells per condition and experiment. Statistical comparison by two-way ANOVA with Šídák’s multiple comparisons test. See also Figures S6–S9.

To address this issue, we first performed a sphere formation assay (SFA) and confirmed that, under our 3D-on top culture conditions, the sphere formation efficiency (SFE) decreased with PKD3 depletion (Figure S6), confirming previous results.^17^ Next, we investigated a potential role of PKD3 in the Wnt pathway and employed the GSK3β inhibitor CHIR99021 to induce canonical Wnt signaling in MDA-MB-231 cells.^45^ As a readout for Wnt activity, we detected non-phosphorylated β-catenin, one of the main effector proteins in the canonical Wnt signaling (reviewed in^48^). Indeed, in control cells, CHIR99021 induced a potent activation of β-catenin, visible by the enrichment of the non-phosphorylated protein. Upon PKD3 depletion, either by the transient transfection of a PKD3-specific siRNA (Figure 4A) or by a stable knockdown (shPKD3#2, Figure 4B), the amount of active β-catenin was significantly reduced compared to control cells. Likewise, when PKD activity was inhibited using the PKD pan-inhibitor CRT0066101 prior to treatment with CHIR99021, a decrease in non-phosphorylated β-catenin was observed (Figure 4C). To further corroborate these data, we conducted qPCR analysis of *Axin2*, widely considered a marker for the Wnt signaling pathway in TNBC.^49^^,^^50^ The depletion of PKD3 either by the transient transfection of PKD3-specific siRNAs or by stably expressed shPRKD3#2, reduced the mRNA levels of *Axin2* in comparison to the control conditions (Figures 4D and 4E). Additionally, both MDA-MB-231 and BT549, another TNBC cell line, showed lower expression of *Axin2* when PKD activity was inhibited by CRT0066101 treatment (Figure S7). Taken together, these results demonstrate an involvement of PKD3 in the Wnt/β-catenin signaling pathway in TNBC cells.

The activation of canonical Wnt signaling via the inhibition of GSK3β has been previously reported to regulate endosomal luminal pH^45^^,^^46^^,^^47^ and to correlate with Rab7 localization to lysosomes.^47^ In fact, a fast drop in the intraluminal pH has been detected during Rab conversion.^51^ For this reason, we next investigated whether PKD3 activity is involved in regulating endosomal acidification. To this end, control and PKD3-depleted MDA-MB-231 (Figure 4F) and BT549 (Figure S8) cells were left untreated or treated with CHIR99021 as previously described and incubated with LysoTracker red. Confocal imaging studies showed a strong and significant increase in LysoTracker dye intensity in control cells treated with the GSK3β inhibitor, confirming previous findings.^47^ Notably, in PKD3-depleted cells, this CHIR99021-induced increase was not visible (Figures 4F and S8), showing that PKD3 is required for the acidification of lysosomes upon the induction of Wnt signaling. We did not detect a significant difference under basal conditions, but a trend toward lower LysoTracker dye intensity for PKD3 depletion was observed in both cell lines.

Additionally, we analyzed the impact of PKD3 activity on acidic vesicles using LysoSensor Yellow/Blue DND-160, which exhibits dual spectral peaks that are pH-dependent. The acidification of the lysosomes detected with the probe was quantified by measuring the intensity of the signal at 451–592 nm, corresponding to the emission spectra in more acidic organelles. As expected, CHIR99021 treatment resulted in a strong increase in fluorescence intensity in control cells, indicating a drop in the luminal pH. Importantly, PKD3-depleted cells failed to acidify the lysosomes in response to the treatment, corroborating our LysoTracker experiments (Figures 4G–4I). Since we were unable to excite the probe in the UV range to detect blue fluorescence, a ratiometric measurement to detect organelles with neutral intraluminal pH was not possible. This could explain why we did not observe significant differences in luminal pH in PKD3-depleted cells under basal conditions, albeit a trend in the LysoTracker experiments was visible. Notably, the Rab7 effector RILP has been reported to regulate the recruitment of ATP6V1G1, a subunit of the lysosomal V-ATPase responsible for acidification, to late endosomal and lysosomal membranes, as well as to control V1G1 stability.^52^ Based on this, we hypothesized that PKD3 depletion might result in V1G1 degradation, thereby impairing lysosomal acidification. However, immunoblot analysis revealed that V1G1 protein levels remained unchanged upon PKD3 knockdown (Figure S9). Thus, PKD3 appears to be required for maintaining endolysosomal acidification in TNBC cells through a mechanism independent of V1G1 abundance.

## Discussion

Our study provides insights into a previously unrecognized localization of endogenous PKD3 to endolysosomes and assigns the kinase a function in the localization of Rab7 to these membranes. Loss of PKD3 leads to a redistribution of Rab7, which affects the localization of the retromer complex and proper lysosomal function.

In our studies, using 3D-on top culture seemed to be key to identifying active PKD3 at endolysosomal vesicles, while its basal activity using standard 2D culture was rather low. This could explain why other studies showed only a few cells where PKD3 was identified in puncta, and these were therefore not fully characterized.^5^^,^^53^ We further demonstrate that endogenous PKD2 localizes to the TGN and does not overlap with endogenous tagged PKD3^EN^-mGFP, supporting an isoform-specific function for PKD3 homodimers at endolysosomes. Notably, PKD2 and PKD3 differ in structure, with PKD3 lacking the postsynaptic density protein-95/disclarge tumor suppressor protein/zonula occludens-1 (PDZ) motif^54^ and both kinases possessing distinct intrinsically disordered regions.^14^ These differences could control specific protein-protein interactions, which in turn, together with DAG, determine the localization of the kinases. Our findings are further in line with a recent study demonstrating a predominant role for PKD2 in regulating the PKD-dependent pro-invasive secretome in metastatic TNBC cells, with only a minor contribution of PKD3.^10^

Furthermore, the here reported defect in acidification upon PKD3 depletion affects the Wnt signaling pathway. Remarkably, *PRKD3* was among the genes up-regulated in Wnt3a-stimulated TNBC cells,^50^ further substantiating a role for the kinase in Wnt signaling. Likewise, by combining proteomics and transcriptomics, Muñoz et al. defined the stem cell signature in the intestine, where the Wnt pathway is one of the major forces controlling homeostasis,^55^ and which included PKD3 among other kinases.^56^ These studies support our hypothesis for a critical role of PKD3 in the Wnt signaling pathway through regulating Rab7 at late endosomes and thus luminal acidification. Our data are consistent with recent findings showing that the PKD activator and tumor promoter phorbolester increased Wnt-driven colorectal cancer progression, suggesting a requirement for lysosomal acidification for Wnt signaling.^57^ In line with the PKD3-dependent localization of Vps35, the retromer complex has also been implicated in Wnt signaling by recycling Wntless from endosomes to the TGN to sustain Wnt secretion.^58^

In MDA-MB-231 cells, Rab7 localization to enlarged endosomes was PKD3-dependent. Rab7 controls endolysosomal function via distinct mechanisms and binds different effectors.^30^^,^^59^ For example, binding to the retromer subunit Vps35 regulates cargo retrieval for hydrolase transport,^32^^,^^33^^,^^38^ while acidification is dependent on the interaction with RILP and the assembly of the V-ATPase.^60^ The defect in acidification observed in PKD3-depleted cells suggests that the Rab7/RILP-dependent recruitment of the ATP6V1G1 (V1G1) subunit of the V-ATPase to late endosomes and/or lysosomes may be perturbed. Notably, this process has been linked to V1G1 stability.^52^ However, PKD3 depletion did not lead to V1G1 degradation, and the lack of commercially available antibodies suitable for immunofluorescence hindered spatial analysis of V1G1 distribution. Furthermore, our data show that PKD3 depletion reduces the co-localization of Rab7 with Vps35, which could underlie the observed defect in CatD trafficking. Disrupted retromer recruitment to endosomes in Rab7-knockout cells has been shown to impair the trafficking of lysosomal sorting receptors, ultimately causing CatD secretion,^31^ a phenotype that mirrors our observations in PKD3-deficient cells. Additionally, impaired endolysosomal acidification may exacerbate this defect, as an acidic environment is required for the dissociation of the mannose-6-phosphate receptor (M6PR) and for proper cleavage and activation of lysosomal hydrolases.^61^ In any case, the presence of active Rab7 on endolysosomal membranes is indispensable for their proper function^30^ and our data place PKD3 upstream of this process.

Within the scope of these studies, a specific PKD3 substrate at endolysosomal membranes has not been identified. Rab7 activity is regulated by the GEF Mon1-Ccz^62^ and different GAP proteins with a conserved Tre2/Bub2/Cdc16 (TBC) domain.^63^ Of these, TBC1D5 is of particular interest since it binds with high affinity to the retromer complex at maturing and late endosomes.^33^^,^^36^^,^^64^^,^^65^ Indeed, recent studies suggest a role for retromer-associated TBC1D5 in regulating the distribution of active Rab7 pools to maintain endolysosomal function,^36^^,^^37^ a role that we show here for PKD3. Our work thus encourages future studies to identify potential PKD3 substrates at endolysosomal membranes, which promote the oncogenic role of lysosomes in TNBC.

### Limitations of the study

Endogenous PKD3 was tagged with mGFP in HeLa cells to study its localization, as tagged MDA-MB-231 cell populations were not stable beyond a few passages. As a result, localization studies in MDA-MB-231 cells relied on the inducible overexpression of PKD3. While this approach allowed the visualization of PKD3-GFP at late endosomal membranes, it does not provide definitive insight into the endogenous localization of PKD3 in this cell line. Moreover, although we demonstrate a role for PKD3 in regulating Rab7 localization and retromer function in TNBC cells, the precise molecular mechanism and direct substrates remain unknown. Phosphoproteomic analyses in combination with protein-proximity labeling techniques will be required to uncover PKD3-specific targets and interaction partners involved in this pathway.

## Resource availability

### Lead contact

Requests for further information and resources should be directed to and will be fulfilled by the lead contact, Angelika Hausser (angelika.hausser@izi.uni-stuttgart.de).

### Materials availability

All unique/stable reagents generated in this study are available from the lead contact with a completed materials transfer agreement.

### Data and code availability

• Data – All data reported in this article will be shared by the lead contact upon request.
• Code –The ImageJ macros created in this study are publicly available at: https://github.com/elena-gutierrez-galindo/Gutierrez-galindo-et-al.
• Other items – Any additional information required to reanalyze the data reported here is available from the lead contact upon request.

## Acknowledgments

We would like to thank Hesso Farhan (Medical University of Innsbruck) for critical discussions and Gisela Link (University of Stuttgart) for technical support. We acknowledge the support from the Cellular Analytics platform of the Stuttgart Research Center Systems Biology (10.13039/501100009534University of Stuttgart, Germany). The graphical abstract was created with BioRender.com. This project has received funding from the European Union’s 10.13039/501100007601Horizon 2020 research and innovation program under the Marie Skłodowska-Curie grant agreement No 859962.

## Author contributions

Conceptualization: E.G.G and A.H.; methodology: E.G.G. and A.H.; formal analysis: E.G.G., K.J., Y.F., and A.H.; software: E.G.G. and F.M.; investigation: E.G.G., K.J, Y.F., and A.H.; data curation: E.G.G.; writing – original draft: E.G.G. and A.H.; writing – review and editing: E.G.G. and A.H.; visualization: E.G.G.; supervision: E.G.G. and A.H.; project administration: A.H.; funding acquisition: A.H.

## Declaration of interests

The authors declare no potential conflict of interest.

## STAR★Methods

### Key resources table

**Table:** 

REAGENT or RESOURCE	SOURCE	IDENTIFIER
**Antibodies**		

Rabbit pAb anti-ATP6V1G1; WB: 1:500	Proteintech	Cat# 16143-1-AP; RRID:AB_2062686
Rabbit mAb anti-Cathepsin D (E5V4H); WB 1:1000	Cell Signaling Technologies	Cat# 74089; RRID:AB_3678646
Mouse mAb anti-CD63 (MEM-259); IF 1:1000	Sigma-Aldrich	Cat# SAB4700215; RRID:AB_10895913
Rabbit pAb anti-GAPDH; WB 1:2000	Sigma-Aldrich	Cat# G9545; RRID:AB_796208
Mouse mAb anti-Lamp1 (D4O1S); IF 1:200	Cell Signaling Technologies	Cat# 15665; RRID:AB_2798750
Rabbit mAb anti-Lamp1 (D2D11); IF 1:200	Cell Signaling Technologies	Cat# 9091; RRID:AB_2687579
Rabbit mAb anti-Non-phospho (Active) β-Catenin (Ser45) (D2U8Y); WB 1:1000	Cell Signaling Technologies	Cat# 19807; RRID:AB_2650576
Rabbit pAb anti-Phospho-PKD/PKCμ (Ser744/748); IF 1:100	Cell Signaling Technologies	Cat# 2054; RRID:AB_2172539
Rabbit mAb anti-PKD2 (D1A7); IF 1:100	Cell Signaling Technologies	Cat# 8188; RRID:AB_10829368
Rabbit mAb anti-PKD3/PKCν (D57E6); WB 1:1000	Cell Signaling Technologies	Cat# 5655; RRID:AB_10695917
Mouse mAb anti-Rab7 (E9O7E); IF 1:100	Cell Signaling Technologies	Cat# 95746; RRID:AB_2800252
Rabbit mAb anti-Rab7 (D95F2); IF 1:50; WB 1:1000	Cell Signaling Technologies	Cat# 9367; RRID:AB_1904103
Sheep pAb anti-TGN46; IF 1:300	Bio-Rad	Cat# AHP500GT; RRID:AB_2203291
Mouse mAb anti ⍺-tubulin (DM1A); WB 1:10000	Merck Millipore	Cat# 05–829; RRID:AB_310035
Rabbit mAb anti-VPS35 (E6S4I); IF1:100, WB 1:1000	Cell Signaling Technologies	Cat# 81453; RRID:AB_2923525
Donkey Anti-Sheep Alexa Fluor™ 546; IF 1:500	Thermo Fisher Scientific	Cat# A-21098; RRID:AB_2535752
Goat anti-Mouse Alexa Fluor™ 488; IF 1:500	Thermo Fisher Scientific	Cat# A-11001; RRID:AB_2534069
Goat anti-Mouse Alexa Fluor™ Plus 647; IF 1:2000	Thermo Fisher Scientific	Cat# A32728; RRID:AB_2633277
Goat anti-Rabbit Alexa Fluor™ 546; IF 1:500	Thermo Fisher Scientific	Cat# A-11010; RRID:AB_2534077
Goat anti-Rabbit Alexa Fluor™ Plus 488; IF 1:2000	Thermo Fisher Scientific	Cat# A32731; RRID:AB_2633280
Goat anti-Rabbit Alexa Fluor™ Plus 555; IF 1:2000	Thermo Fisher Scientific	Cat# A32732; RRID:AB_2633281
Goat anti-Rabbit Alexa Fluor™ Plus 647; IF 1:2000	Thermo Fisher Scientific	Cat# A32733; RRID:AB_2633282
Goat anti-Mouse Alexa Fluor™ Plus 405; IF 1:2500	Thermo Fisher Scientific	Cat# A48255; RRID:AB_2890536
Peroxidase AffiniPure™ Goat Anti-Mouse IgG (H + L); WB 1:10000	Jackson Immunoresearch	Cat# 115-035-062; RRID:AB_2338504
Peroxidase AffiniPure™ Goat Anti-Rabbit IgG (H + L); WB 1:10000	Jackson Immunoresearch	Cat# 111-035-144; RRID:AB_2307391

**Bacterial strains**		

*Escherichia coli* DH5α	Thermo Fisher	Cat# 18265017

**Chemicals, peptides, and recombinant proteins**		

CHIR99021	Tocris Bioscience	Cat# 4423
CRT0066101	Tocris Bioscience	Cat# 4975
LysoTracker DND-99 Red	Invitrogen	Cat# L7528
LysoSensor Yellow/Blue DND-160	Invitrogen	Cat# L7545

**Experimental models: Cell lines**		

MDA-MB-231	CLS	RRID:CVCL_0062
MDA-MB-231_shNTC#2	P. Storz, Mayo Clinic, Florida (Borges et al.)^16^	N/A
MDA-MB-231_shPRKD3#2	P. Storz, Mayo Clinic, Florida (Borges et al.)^16^	N/A
MDA-MB-231_PKD3-GFP	This study	N/A
BT549	CLS	RRID:CVCL_1092
HeLa	ATCC	RRID:CVCL_0030
HeLa_PKD3^EN^-mGFP	This paper	N/A
LentiX HEK293T	Dr. Philipp Rathert, University of Stuttgart	N/A

**Oligonucleotides**		

Oligonucleotides used for sequencing and cloning: see Table S1	N/A	N/A
siNTC (ON-TARGETplus® non-targeting control pool)	Horizon Discovery	Cat# D-001810-10
spPRKD3#1 (ON-TARGETplus Human PRKD3 siRNA - set of 4)	Horizon Discovery	Cat# LQ-005029-00

**Recombinant DNA**		

pCW57-MCS1-P2A-MCS2 (Neo)	Adam Karpf, University of Nebraska Medical Center	Addgene plasmid #89180
psPAX2	Didier Trono, École Polytechnique Fédérale de Lausanne	Addgene plasmid #12260
pCMV-V-SVG	(Stewart et al.)^66^	Addgene plasmid #8454
pCW57-MCS1-P2A-MCS2-PKD3-GFP	This paper	N/A
pEGFP-N1-PKD3	(Huck et al.)^5^	N/A
pMaCTag-P05	(Fueller et al.)^24^	Addgene plasmid #120016
pCAG-enAsCas12a(E174R/S542R/K548R)-NLS(nuc)-3xHA (AAS848)	(Kleinstiver et al.)^67^	Addgene plasmid #107941
pGEX-4T-3-mR7BD	(Romero Rosales et al.)^68^	Addgene plasmid #79149

**Software and algorithms**		

Fiji	https://imagej.net/software/fiji/	RRID:SCR_002285
GraphPad Prism (Version 10)	GraphPad Software Inc.	RRID:SCR_002798
Zeiss Zen Lite	Carl Zeiss	RRID:SCR_023747
Zeiss ZEN (blue edition)	Carl Zeiss	RRID:SCR_013672
ImageJ macros created in this study	https://github.com/elena-gutierrez-galindo/Gutierrez-galindo-et-al.	N/A

### Experimental model and study participant details

#### Cell lines

The TNBC cell lines MDA-MB-231, BT549, MDA-MB-231-based knockdown cells (in text: shNTC#2 and shPRKD3#2^16^), MDA-MB-231_PKD3-GFP and HeLa_PKD3^EN^-mGFP (both generated in this study) were cultured in high-glucose Dulbecco’s modified Eagle’s medium (DMEM, Gibco). HeLa cells were cultured in RPMI-1640 (Gibco). All cell lines were supplemented with 10% Fetal Bovine Serum (FBS, Sigma #F7524), cultured at 37 °C in a humidified chamber with 5% CO_2_, and maintained until 80–90% confluence prior passaging, for no longer than 2 months. All cell lines used in this study were authenticated by SNP profiling (Multiplexion GmbH), and confirmed free of Mycoplasma contamination (Lonza). MDA-MB-231_PKD3-GFP cells were maintained with 500 μg/mL G418 sulfate (Geneticin, Calbiochem #345810) and HeLa_PKD3^EN^-mGFP with 2 μg/mL Puromycin (Thermo Fisher Scientific).

### Method details

#### Generation of MDA-MB-231_PKD3-GFP cells

MDA-MB-231_PKD3-GFP cells were generated by lentiviral transduction. The PKD3-GFP encoding module was subcloned from pEGFP-N1-PKD3^5^ into the vector pCW57-MCS1-P2A-MCS2 (*Neo*) (a gift from Adam Karpf, Addgene #89180) by AgeI restriction using the NEBuilder HiFi DNA Assembly Cloning Kit (New England Biolabs #E55220). Lentiviral particles were generated by cotransfecting LentiX HEK293T cells (provided by Dr. Philipp Rathert, University of Stuttgart, and maintained in DMEM with 10% FBS) with virus packaging vectors psPAX2 (a gift from Didier Trono, Addgene #12260), pCMV-V-SVG (a gift from Bob Weinberg, Addgene #8454^66^) and pCW57-MCS1-P2A-MCS2-PKD3-GFP. Transfection was performed using a 1:3 (w/w) mixture of DNA to polyethylenimine (Sigma Aldrich). The resulting virus soup was collected and filtered 24 h after transfection and used for transduction of MDA-MB-231 cells in combination with 8 μg/mL polybrene (Millipore). The transfected cells were selected with 500 μg/mL G418 sulfate. The successful expression of PKD3-GFP was assessed by immunofluorescence and Western blot. During the experiments, PKD3-GFP expression was induced with 1 μg/mL doxycycline for the last 24 h of the experiment.

#### CRISPR/Cas12a assisted PCR tagging

Endogenous PKD3 was tagged at the C-terminus using a PCR-based CRISPR-Cas12a approach.^24^ A modified plasmid pMaCTag-P05 (a gift from Michael Knop, Addgene #120016^24^), harboring the monomerizing A206K mutation in EGFP, was used as PCR template with the oligos M1_PKD3 and M2_PKD3 (Table S1). Cells were transfected with purified PCR product and enAsCas12a helper plasmid (a gift from Keith Joung and Benjamin Kleinstiver; Addgene #107941^67^) using Lipofectamine 2000 (Thermo Fisher #11668019). Three days after transfection puromycin was added at a concentration of 2 μg/mL.

#### Transient transfection

For reverse transient transfection, 2 x 10^5^ cells were seeded into 6-well plates and transfected with 15 pmol of a mix of four individual siRNAs (Non-Targeting Control or Human *PRKD3*; in text, spNTC#1 or spPRKD3#1; ON-TARGETplus SMARTpool, Horizon Discovery), using Lipofectamine RNAiMAX Transfection Reagent (Invitrogen #13778150) for TNBC cell lines and TransIT-HeLaMONSTER Transfection Kit (Mirus Bio #MIR2904) for HeLa and HeLa_PKD3^EN^-mGFP cells.

#### Genomic DNA isolation and PCR

Genomic DNA was isolated from HeLa_PKD3^EN^-mGFP cells using the PureLink Genomic DNA Kit (Thermo Fisher #K182001) according to the manufacturer’s instruction. The C-terminal region of the *PRKD3* gene was amplified from genomic DNA by PCR using the primers GRCh38.p1471479 For and GFP SalI Rev (Table S1). After purification, the PCR product was sequenced using the primer GRCh38.p1471479 for.

#### Preparation of polyacrylamide (PAA) hydrogels

Polyacrylamide (PAA) hydrogels were prepared based on the protocol previously described.^69^ Briefly, 18- or 24-mm square No. 1.5, or 42 mm circle coverslips were incubated with 0.1 M NaOH for 5 min, dried, incubated with 3-Aminopropyltriethoxysilane (APTES, Sigma #440140) for 3 min, washed with ddH_2_O for 30 min, dried, incubated with 0.5% glutaraldehyde (Sigma #G7651) in PBS for 30 min and dried. A mix of acrylamide and bis-acrylamide (BioRad #1610140, #1610142) was prepared according to desired stiffness (5 kPa) based on the publication above and polymerized with ammonium persulfate (APS) and Tetramethylethylenediamine (TEMED, Roth #2367). The mixture was polymerized between the amino-silanated coverslip and a Dichlorodimethylsilane (DCDMS, PlusOne GE Healthcare #17-1332-01)-coated coverslip for 15 min. After removing the top coverslip, the hydrogels were rinsed twice with PBS and stored at 4 °C. Prior use, hydrogels were functionalized with 0.2 mg/mL sulfo-SANPAH (Thermo Scientific, #22589), diluted HEPES buffer (50 mM, pH 8.5) under 365 nm UV light for 20 min, washed twice with HEPES buffer and incubated overnight at 4 °C with 100 μg/mL PureCol Type I Collagen Solution Bovine (Advanced Biomatrix #5005).

#### 2D culture

Experiments with HeLa and HeLa_PKD3^EN^-mGFP cells were performed using 2D culture. Cells were cultured on tissue-culture plates or on glass coverslips coated with 2.5 μg/mL Collagen R Solution (Serva #47254) for 1 h at 37 °C or overnight at 4 °C. When transient transfection was performed, cells were fixed or harvested 72 h post-transfection.

#### 3D-on top culture

Unless indicated, all experiments with TNBC cell lines were performed using 3D-on top culture. Transient transfection (when applicable) was performed 16–24 h prior harvesting and re-seeding cells on hydrogels.

This protocol was adapted from that described elsewhere.^20^ Collagen-coated PAA hydrogels were prepared as described above, rinsed with PBS and UV-sterilized for 20 min. TNBC cell lines were collected by trypsinization, and the appropriate cell count was resuspended in ice-cold DMEM-high glucose supplemented with 10% FBS, 1% Penicillin/Streptomycin and 2% Matrigel Matrix (Corning #356231), plated on hydrogels and incubated for 5 days.

#### Sphere formation assay

TNBC cells were seeded on 5 kPa PAA hydrogels using the protocol for 3D-on top described above. After 5 days, cells were collected by trypsinization, singularized using a 27G needle, resuspended in sphere formation medium (DMEM/F12 supplemented with 10 μg/mL insulin (Sigma-Aldrich #I6634), 20 ng/mL EGF (R&D Systems #236-EG-200), 1 μg/mL hydrocortisone (Sigma-Aldrich #HO888), 1x B27-supplement (Gibco #17504044) and 1% Penicillin/Streptomycin), seeded onto Poly(2-hydroxyethyl methacrylate)-(pHEMA, Sigma-Aldrich)-coated plates in triplicates and incubated for 5 days. 3 x 10^3^ cells were seeded in 1.5 mL/well in a 12-well plate. Spheres were imaged on day 5 and analyzed using a semiautomated macro in *Fiji*. First, the spheres were outlined using the function *Sharpen* and *Find edges*, then thresholded and converted to binary images. Holes and borders were closed using 3 iterations of the functions *Erode* and *Dilate*, and the area was quantified using *Analyze particles*. Stemness was evaluated by the sphere formation efficiency (SFE, number of spheres formed per 1000 cells seeded). Only spheres > 2500 μm^2^ were considered.

#### Western blot

Whole-cell lysates were prepared in RIPA buffer (50 mM Tris pH 7.4, 150 mM NaCl, 1 mM EDTA, 1% NP40, 0.25% sodium deoxycholate, 0.1% SDS) with phosphatase (PhosSTOP, Roche) and protease inhibitors (cOmplete EDTA-free, Roche), clarified by centrifugation at 13000 rpm for 15 min and quantified using the DC Protein Assay Kit (Bio-Rad #5000112). Equal amounts of protein were loaded, separated by SDS-page (NuPAGE, Invitrogen) and transferred into a nitrocellulose membrane using an iBlot gel transfer device and iBlot Transfer Stacks (both from Invitrogen). The membranes were incubated with Blocking solution (Roche #11096176001) in PBS containing 0.05% Tween-20 and the antibodies as indicated, diluted in Blocking solution. Acquisition was done using an Amersham Imager 600 device (Cytiva) and the respective band intensities were quantified in *Fiji* using the raw images and normalized to the loading control (α-tubulin or GAPDH) and to the average intensity for each independent experiment. Figures show a representative membrane after auto-contrast adjustment. All original blots are available as DataSource.

#### GST-RILP production and pull-down

The purchased glycerol stock from *E. coli* transformed with the plasmid containing the nucleotides 658–897 of the murine Rab-interacting lysosomal protein (RILP) protein, fused to the C-terminus of GST in the pGEX 4T-3 vector (pGEX-4T-3-mR7BD, a gift from Aimee Edinger, Addgene #79149^68^), was inoculated in 5 mL of LB medium with Ampicillin. Then, 1 mL of the overnight culture was used to inoculate 250 mL of LB medium with Ampicillin and grown at 37 °C to an optical density (OD) of 0.65. Then, IPTG was added to a final concentration of 0.5 mM to induce protein production, and the culture was incubated for additional 4 h at 30 °C, then spun down at 5000 rpm for 10 min, after which the supernatant was discarded, and the bacteria pellet frozen at 20 °C. The bacteria were lysed in 5 mL of cold bacteria lysis buffer (25 mM Tris-HCl pH 7.4, 1M NaCl, 0.5 mM EDTA, 0.1% Triton X-100, 1 mM DTT) with complete protease inhibitors and sonicated in 3 cycles of 30 s using a Sonoplus ultrasonic homogenizer. Then the lysates were cleared by centrifugation for 10 min at 13000 rpm. Subsequently, 5 mL of lysis buffer were added to the cleared lysate prior incubation with 300 μL of a pre-equilibrated 50% slurry of glutathione Sepharose 4B beads for 1 h at 4 °C with continuous rotation. The beads were centrifuged at 1500 rpm for 3 min, washed twice with lysis buffer and resuspended as a 50% slurry in lysis buffer. An estimation of the concentration as well as verification of the correct binding was performed by SDS-PAGE and subsequent Coomassie staining.

For the pull-down assays, whole-cell lysates were prepared using 500 μL of lysis buffer (10 mM HEPES, 150 mM NaCl, 5 mM MgCl2, 5% Glycerol, 0.1% NP-40, 1 mM DTT) with phosphatase (PhosSTOP, Roche) and protease inhibitors (cOmplete EDTA-free, Roche) and passed through a 25G needle ten times to solubilize membrane-bound proteins. After 5 min incubation on ice, lysates were clarified by centrifugation at 13000 rpm for 15 min. 50 μL were kept as input and the rest was incubated in a final volume of 1 mL lysis buffer with 10 μL of a 50% slurry GST-RILP beads coupled to glutathione Sepharose 4B beads, for 1 h at 4 °C with continuous rotation, spun down and washed thrice with washing buffer (10 mM HEPES, 150 mM NaCl, 5 mM MgCl2, 5% Glycerol, 1 mM DTT). The supernatant was removed completely, and the beads were eluted by boiling in 5x Laemmli-sample buffer for 10 min at 96 °C.

#### Co-immunoprecipitation

At 72 h post-transient transfection, MDA-MB-231 cells cultured on tissue culture dishes were lysed on ice for 15 min using ice-cold lysis buffer (0.5% NP40, 10 mM Tris pH 7.5, 150 mM NaCl, 0.5 mM EDTA). The lysates were then centrifuged for 10 min at 16000 × g and 4 °C. 10% of the cleared lysate was reserved as an input sample, while the remaining 90% was used for immunoprecipitation. The pre-cleared lysate was first incubated with a Rab7 rabbit monoclonal antibody for 1.5 h at 4°C, followed by incubation with protein G agarose beads for 1 h at 4°C under end-over-end rotation. Beads were washed three times with lysis buffer to remove non-specifically bound proteins. Bound proteins were eluted with sample buffer and analyzed by SDS-PAGE and Western blotting as described above.

#### Cathepsin D secretion assay

The cathepsin D secretion assay was performed as previously described.^70^ Briefly, 72 h post-transfection, MDA-MB-231 cells transfected with specific siRNAs and cultured on tissue culture dishes were incubated in serum-free medium and chased for 4.5 h. The medium was then collected and proteins were precipitated using 20% trichloroacetic acid. The resulting pellet was resuspended in 5x Laemmli sample buffer. Cells were harvested and lysed (150 mM NaCl, 50 mM Tris-HCl pH 7.5, 2 mM EDTA, 1% Triton X-100, and protease and phosphatase inhibitor cocktail) for 30 min on ice. Both the precipitated proteins from the medium and the cell lysates were analyzed by Western blotting.

#### Quantitative real-time PCR (qRT-PCR) analysis

RNA was isolated from cells using NucleoSpin RNA kit (Macherey-Nagel, #740955). 80 ng per sample were loaded and one-step RT-PCR reaction was performed in triplicates using Power SYBR Green RNA-to-C_T_™ 1-Step Kit (Applied Biosystems, #4389986). The following primers were used: *Axin2*, *PRKD3* (QuantiTect Primer Assay, Hs_PRKD3_1_SG, NM_005813) and *RPLP0* (Table S1). Analysis was performed using the CFX96 Touch Real-Time PCR Detection System (Bio-RAD). mRNA expression values were generated using ΔCt values normalized to *RPLP0*.

#### Immunofluorescence and confocal imaging

Cells were washed with PBS, fixed with 4% paraformaldehyde (PFA) for 20 min at room temperature, permeabilized with PBS-0.1% Triton X-100 for 5 min, and incubated with blocking buffer (PBS-5% FBS). Samples were incubated with primary antibodies overnight in blocking buffer, washed 3 times with PBS, incubated with secondary antibodies with or without 1 μg/mL DAPI for 1 h at room temperature, washed 3 times with PBS and embedded in ProLong Gold Antifade Mountant (Invitrogen #P36930). All samples were analyzed at room temperature using a confocal laser scanning microscope (LSM 980 Airyscan 2) equipped with an LD LCI Plan Apochromat 40x/1.2 Imm Corr DIC M27 water-immersion objective for cells cultured on hydrogels, or with a Plan-Apochromat 63×/1.40 DIC M27 for cells cultured on glass coverslips (both from Carl Zeiss). The respective laser excitation wavelengths and emission detection intervals were used as follows: 653 nm and 574–720 nm for Alexa 647+; 557 nm and 380–608 nm for Alexa Fluor 546 or Alexa Fluor 555+; 493 nm and 499–548 nm for Alexa Fluor 488, Alexa Fluor 488+ or GFP and 353 nm and 422–477 nm for DAPI. Images were acquired in Z-stacks of 250 nm intervals throughout the cell and processed using *ZEN blue* software (Carl Zeiss). Each set of samples was acquired using the same laser settings. For analysis, a single middle Z-section or maximum intensity projections (MIP) were used as indicated in the figure legends. Images were further analyzed in *Fiji* or *ZEN lite* software as described below.

#### Endolysosomal acidification

Cells cultured on 5 kPa hydrogels were incubated with 2 μM LysoSensor Yellow/Blue DND-160 (Thermo Fisher Scientific #L7545) for 5 min prior washing, fixation and mounting as described above. To detect variances in the more acidic lysosomes, the probe was excited at 405 nm and the emission spectra was detected at 451, 459, 468, 477, 486, 495, 504, 512, 521, 530, 539, 556, 565, 574, 583 and 592 nm of a single middle Z-section. For quantification, images were randomized and the mean fluorescence intensity (MFI) of at least 5 vesicles per cell was evaluated using *ZEN lite*. The background was subtracted using the signal in the cell nucleus. An average fluorescence per cell normalized to the average intensity at 451 nm was used to create histograms for each individual experiment, and statistical analysis was performed using the area under the curve (AUC), in three independent experiments.

#### Image analysis

The analysis of immunofluorescence images was performed using *Fiji* or *ZEN lite* softwares. The macros used for quantification in *Fiji* are available at https://github.com/elena-gutierrez-galindo/Gutierrez-galindo-et-al.

*2D* vs*. 3D-on top PKD3-GFP vesicles* (Figure 1B)*:* Maximum intensity projections (MIPs) were used for this quantification. The cell outline was drawn using the *Freehand selection* tool in the GFP channel with increased brightness, and the background was cleared. For each cell, the GFP and pPKD channels were split and a threshold was set for each channel to segment the respective vesicles and converted to a binary image. Then, using the function *Colocalization threshold*, the area between the two channels was segmented and further thresholded into a new binary image, and the pPKD-PKD3 vesicle area was obtained with the *Analyze particles* tool.

*PKD3*^*EN*^*-mGFP vesicles* (Figures 1H and S4C)*:* MIPs were used for this quantification. The cell outline was drawn, and the background was cleared as described. For each cell, the GFP signal was smoothed using the *Gaussian blur* filter and the PKD3^EN^-mGFP vesicles were detected and counted using the *Find maxima* tool. Of note, this function always finds at least one local maxima, so PKD3^EN^-mGFP count per cell is at least 1, even when no clear vesicles are visible.

*Rab7/Vps35 intensity and distribution:* MIPs were used for this quantification. The cell outline was drawn, and the background was cleared as described. The Rab7 or Vps35 MFI in the whole cell was obtained. Additionally, for Rab7, the signal noise was reduced using the function *Subtract background*, and the Rab7 vesicles were identified and saved as ROIs using the plugin *TrackMate*.^71^ Finally, the ROIs were opened for each cell, the cytosolic background was subtracted, and the same threshold was set for all experiments to segment the Rab7-positive vesicles, then they were converted to binary images and measured using the *Analyze particles* tool.

*Colocalization of Rab7 or PKD3*^*EN*^*-mGFP with endosomal/Golgi markers:* The cell outline was drawn, and the background was cleared as described. The Lamp1, CD63 and Vps35 vesicles or TGN46 compartment were segmented for each individual Z-section and saved as ROIs. Colocalization of Rab7 or PKD3^EN^-mGFP with these proteins was quantified as the MFI of Rab7 or PKD3^EN^-mGFP measured for these ROIs and plotted as average intensity per cell.

### Quantification and statistical analysis

Data are presented as mean ± SD. Statistical analysis was performed using the software *GraphPad Prism 10*. Significance between two groups was assessed by t-test using two-tailed unpaired analysis. In studies combining knockdown and treatment, *p*-values were obtained by two-way ANOVA with Šídák’s multiple comparisons test, between control and knockdown for each treatment or between treatments for each siRNA/shRNA group. In studies with different treatments, a one-way ANOVA with Dunn’s multiple comparisons test was used. For confocal microscopy experiments, superplots were used to visualize quantification results, following previously described guidelines.^72^ Small data points on the superplot represent the average per cell, and the bigger data points represent the mean value per experiment, used for statistical analysis. Unless indicated, all experiments were performed at least thrice. Symbols shape in graphs represent independent experiments. The sample size (*N*), replicates (*n*) and statistical test are indicated in the figure legends.

## Supplemental information

Document S1. Figures S1–S9 and Table S1

Data S1. Uncropped Western blots, related to Figures 1, 2, 3, 4, S1, and S2
