# Supplementary material for: PKD3 localizes to late endosomes to maintain Rab7-dependent endolysosomal homeostasis
Source: iScience. 2025 Aug 20;28(9):113408. doi: 10.1016/j.isci.2025.113408 (PMC12441718; doi:10.1016/j.isci.2025.113408)
Supplement: Document S1. Figures S1–S9 and Table S1 [file mmc1.pdf]

iScience, Volume *28*

Supplemental information

PKD3 localizes to late endosomes to maintain Rab7-dependent endolysosomal homeostasis

Elena Gutiérrez-Galindo, Katharina Jursik, Yannick Frey, Florian Meyer, and Angelika Hausser

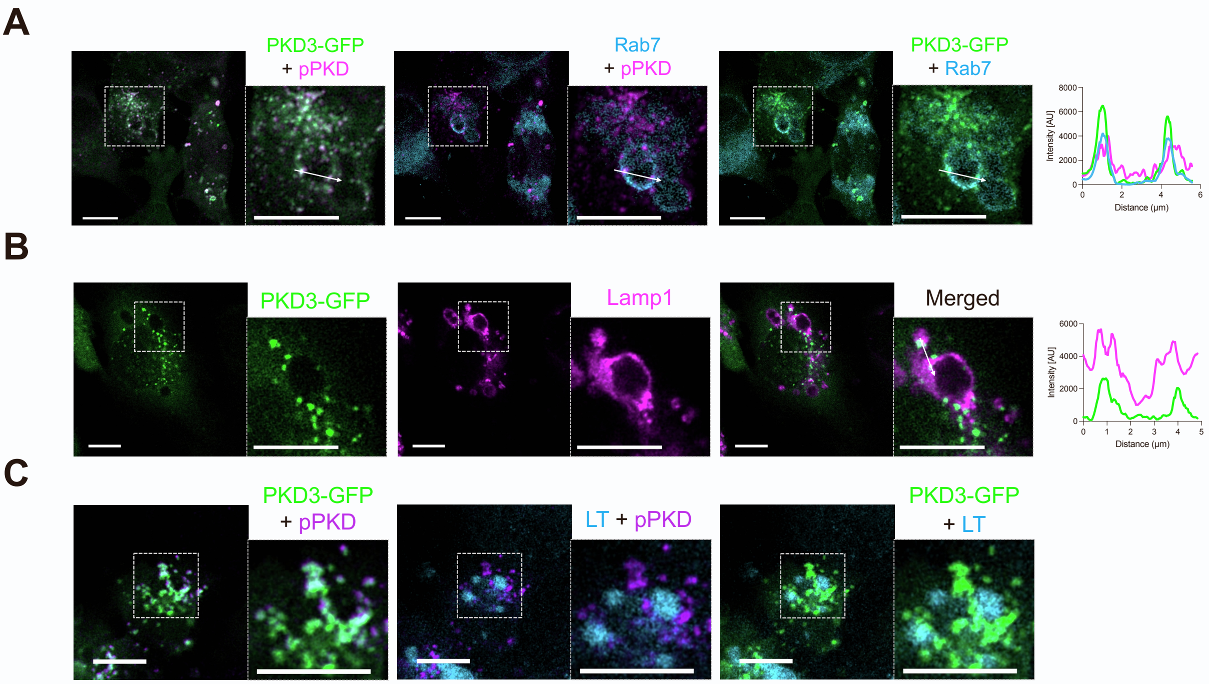

Figure S1. Active PKD3-GFP co-localizes with endolysosomes; Related to Figure 1. MDA-MB-231_PKD3-GFP cells were seeded on 5 kPa PAA hydrogels, incubated with LysoTracker (in C, blue), fixed and stained for different markers as indicated: Rab7 (blue) in **A**, Lamp1 (magenta) in **B** and pPKD (magenta) in **A** and **C**; a middle Z-section is shown; scale bar: 10 µm; the histogram represents the intensity profile in the area marked with a white arrow in the merged image.

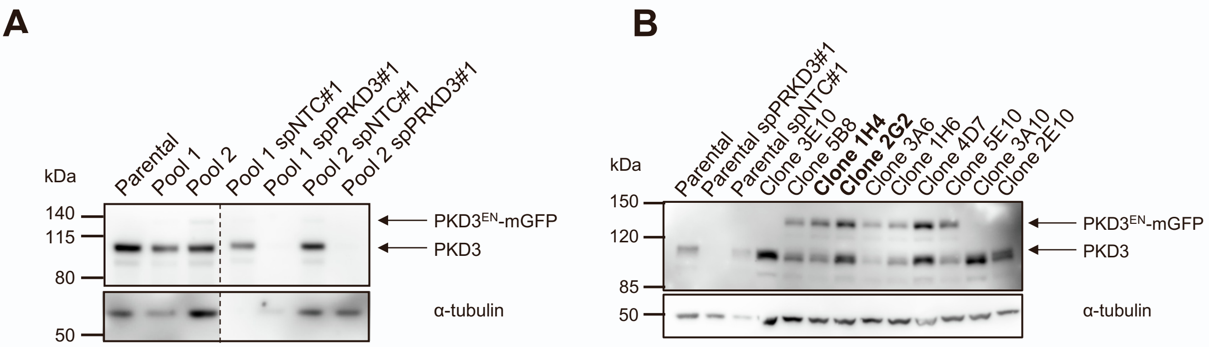

Figure S2. Screening and selection of PKD3^{EN}-mGFP tagged HeLa clones; Related to Figure 1. A) Parental HeLa cells and two different pools of HeLa_PKD3^{EN}-mGFP cells were seeded and cells from Pool 2 were transiently transfected with spNTC#1 or spPRKD3#1. Whole cell lysates were subjected to Western blot analysis and probed for PKD3; α-tubulin was used as a loading control. Dashed line indicates the position where samples of Pool 1 (sorted) and Pool 2 (sorted) were cropped from the same blot. **B)** Parental HeLa cells were seeded and transfected with spNTC#1 or spPRKD3#1. 10 different clones resulting from the limited dilution of Pool 2 of HeLa_PKD3^{EN}-mGFP were seeded. Whole cell lysates were subjected to Western blot analysis and probed for PKD3; α-tubulin was used as a loading control. Positive clones selected for further analysis are labelled in bold.

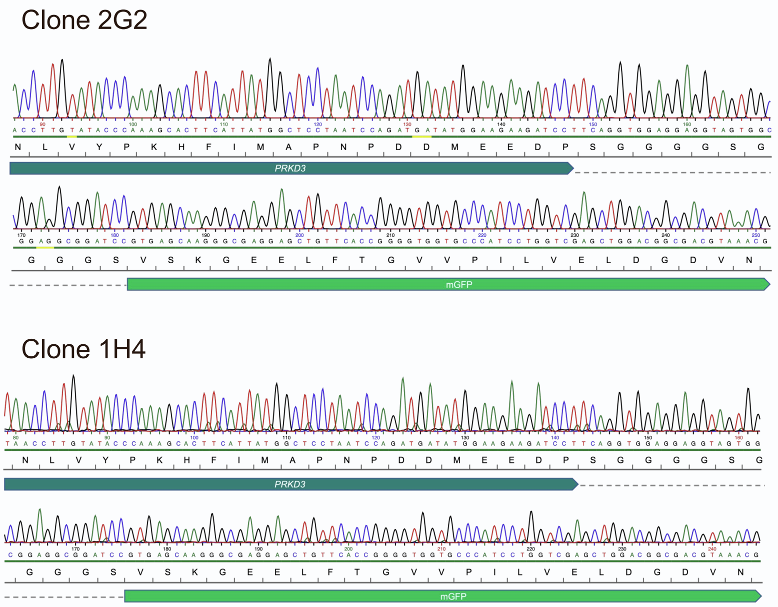

Figure S3. Genomic DNA sequencing of HeLa_PKD3^{EN}-mGFP clones 2G2 and 1H4; Related to Figure 1. The amino acids corresponding to the C-terminus of PKD3, the linker and mGFP are indicated.

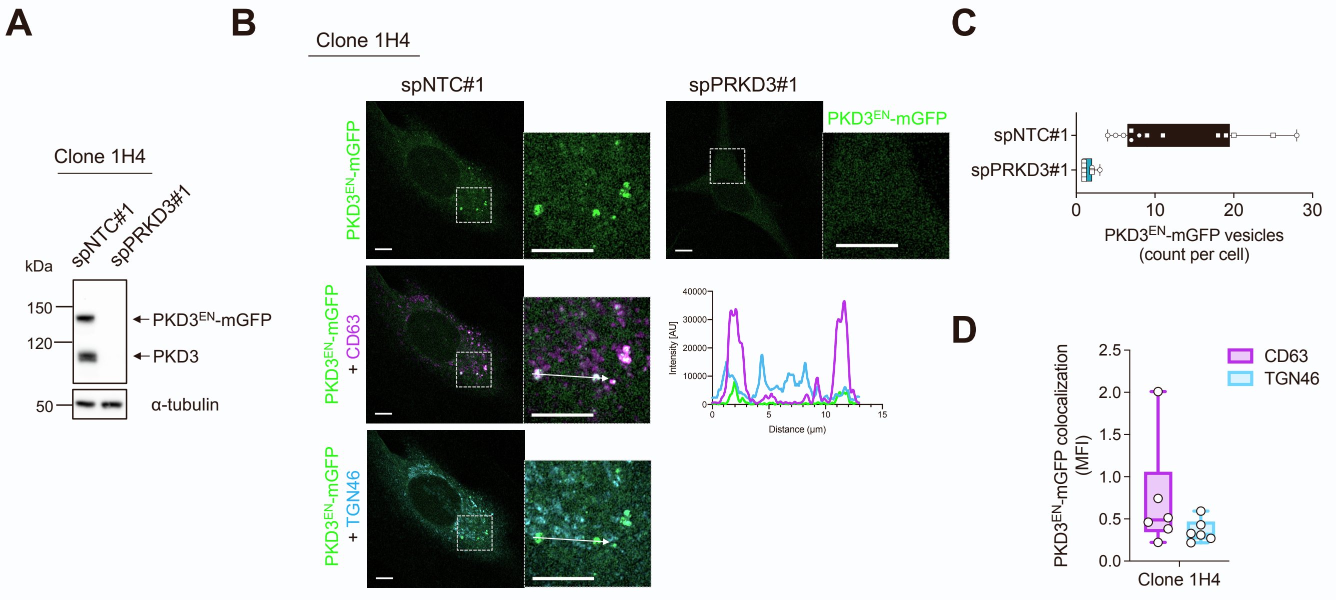

Figure S4. Endogenous tagging of PKD3 with mGFP validated by siRNA knockdown; Related to Figure 1. A) Whole cell lysates of HeLa_PKD3^{EN}-mGFP (Clone 1H4) transiently transfected with spNTC#1 or spPRKD3#1 were subjected to Western blot analysis and probed for PKD3; α-tubulin was used as a loading control. **B)** HeLa_PKD3^{EN}-mGFP cells (Clone 1H4) transiently transfected with spNTC#1 or spPRKD3#1 were seeded on glass coverslips and fixed; control samples were stained for CD63 (magenta) and TGN46 (blue); scale bar: 10 μm; the histogram represents the intensity profile in the area marked with a white arrow. **C-D)** Quantification of B). **C)** the graph represents the number of PKD3^{EN}-mGFP positive vesicles per cell; N > 10; n=2. **D)** Colocalization analysis of PKD3^{EN}-mGFP with CD63 and TGN46, analised as the MFI of PKD3EN-mGFP on CD63- or TGN46-positive vesicles; n=1.

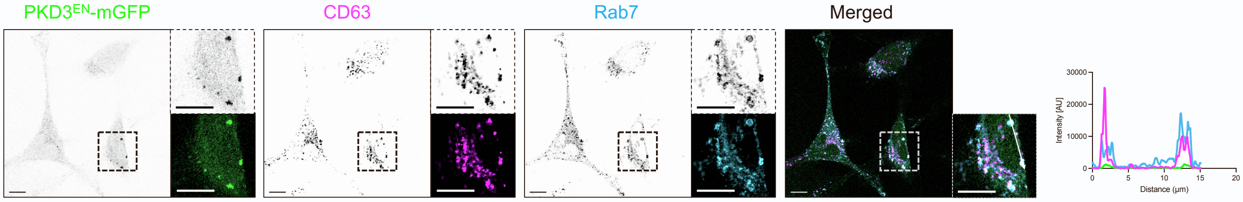

Figure S5. Endogenous PKD3^{EN}-mGFP localizes to CD63 and Rab7-positive vesicles; Related to Figure 1. HeLa_PKD3^{EN}-mGFP cells (Clone 1H4) were seeded on glass coverslips, fixed and stained for CD63 (magenta) and Rab7 (blue); scale bar: 10 µm; the histogram represents the intensity profile in the area marked with a white arrow in the merged image.

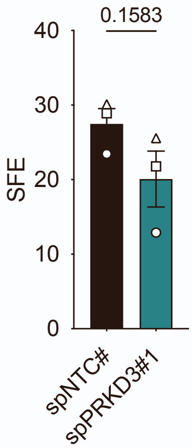

Figure S6. PKD3 loss reduces sphere formation efficency in 3D-on top culture; Related to Figure 4. Quantification of SFA of MDA-MB-231 cells, shown as the sphere formation efficiency (SFE); n=3; statistical comparison by t-test.

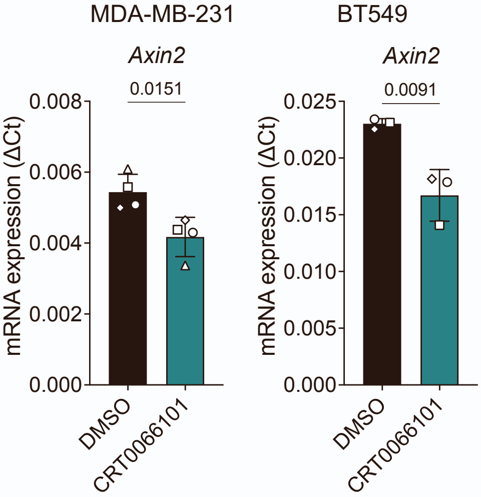

Figure S7. PKD inhibition reduces *Axin2* expression; Related to Figure 4. qPCR analysis of *Axin2* and *PRKD3* in cells seeded on 5 kPa PAA hydrogels; MDA-MB-231 (left) or BT549 (right) were treated with DMSO or CRT0066101 (1 µM) overnight; n= 3-4; statistical comparison by unpaired t-test.

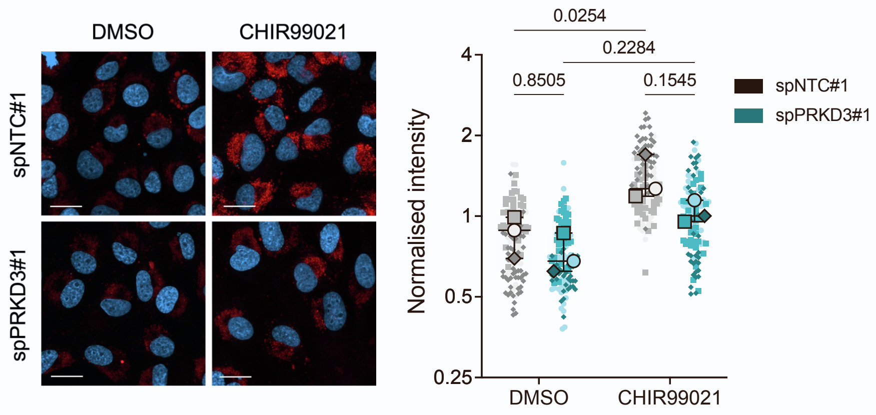

Figure S8. PKD3 supports endolysosomal acidification; Related to Figure 4. BT549 cells transiently transfected with spNTC#1 or spPRKD3#1 were seeded on 5 kPa PAA hydrogels, treated with DMSO or CHIR99021 (8 nM) for 2 h, incubated with LysoTracker and fixed; scale bar: 20 µm; data in graph represents the MFI, normalized to the average MFI for each independent experiment; statistical comparison by two-way ANOVA with Šídák's multiple comparisons test; n= 3, N > 35 cells per condition and experiment.

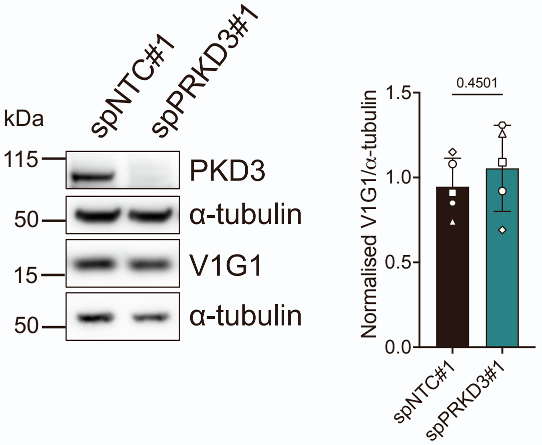

Figure S9. V1G1 levels remain stable upon PKD3 depletion; Related to Figure 4. Whole cell lysates of MDA-MB-231 cells transiently transfected with spNTC#1 or spPRKD3#1 were subjected to Western blot analysis and probed for PKD3 and V1G1; α-tubulin was used as a loading control. The α-tubulin and PKD3 blots were reused from Figure 3E, as they originate from the same experimental samples. The blots are representative of five independent experiments, and the graph shows the quantification; statistical comparison by unpaired t-test.

Plasmid	Sequence
AXIN2	F: 5'- AGT GTC TCT ACC TCA TTT CCC -3'<br>R: 5'- CCC TCT CTC TCT TCA TCC TC -3'
GFP SalI	R: 5′- AAA GTC GAC GCC TTG TAC AGC TCG TCC ATG CC -3′
GRCh38.p1471479	F: 5′- CCT AGG ACT ATC AGA CTTG GCT -3′
M1_PKD3	5′- CGC TGG GAA ATA CAT GCA TAC ACA CAT AAC CTT GTA TAC CCA AAG CAC TTC ATT ATG GCT CCT AAT CCA GAT GAT ATG GAA GAA GAT CCT TCA GGT GGA GGA GGT AGT G -3′
M2_PKD3	5′- AGC AAA ATA TCA GTC CAT AAA ATG AAA TCC TTC CTT ATT TAG GTT AGC TCA GTG AAA AAA AAG TGA TTA AGG ATC TTC TTC ATC TAC AAG AGT AGA AAT TAG CTA GCT GCA TCG GTA CC -3′
PKD3-GFP	F: 5'- GGC TAG CGA ATT CGT CGA CAG CCA CCA TGT CTG CAA ATA ATT CCC C -3'<br>R: 5'- AGA AGT TCG TGG CTC CGG AAA CTT GTA CAG CTC GTC CAT G -3'
PRKD3	QuantiTect Primer Assay, Hs_PRKD3_1_SG, NM_005813
RPLP0	F: 5'- CTC TGC ATT CTC GCT TCC TGG AG -3'<br>R: 5'- CAG ATG GAT CAG CCA AGA AGG -3'

Table S1. List of oligonucleotides used for sequencing and cloning; Related to STAR Methods.
