# Supplementary material for: PKD3 localizes to late endosomes to maintain Rab7-dependent endolysosomal homeostasis
Source: iScience. 2025 Aug 20;28(9):113408. doi: 10.1016/j.isci.2025.113408 (PMC12441718; doi:10.1016/j.isci.2025.113408)

Data Source – Figure S1D

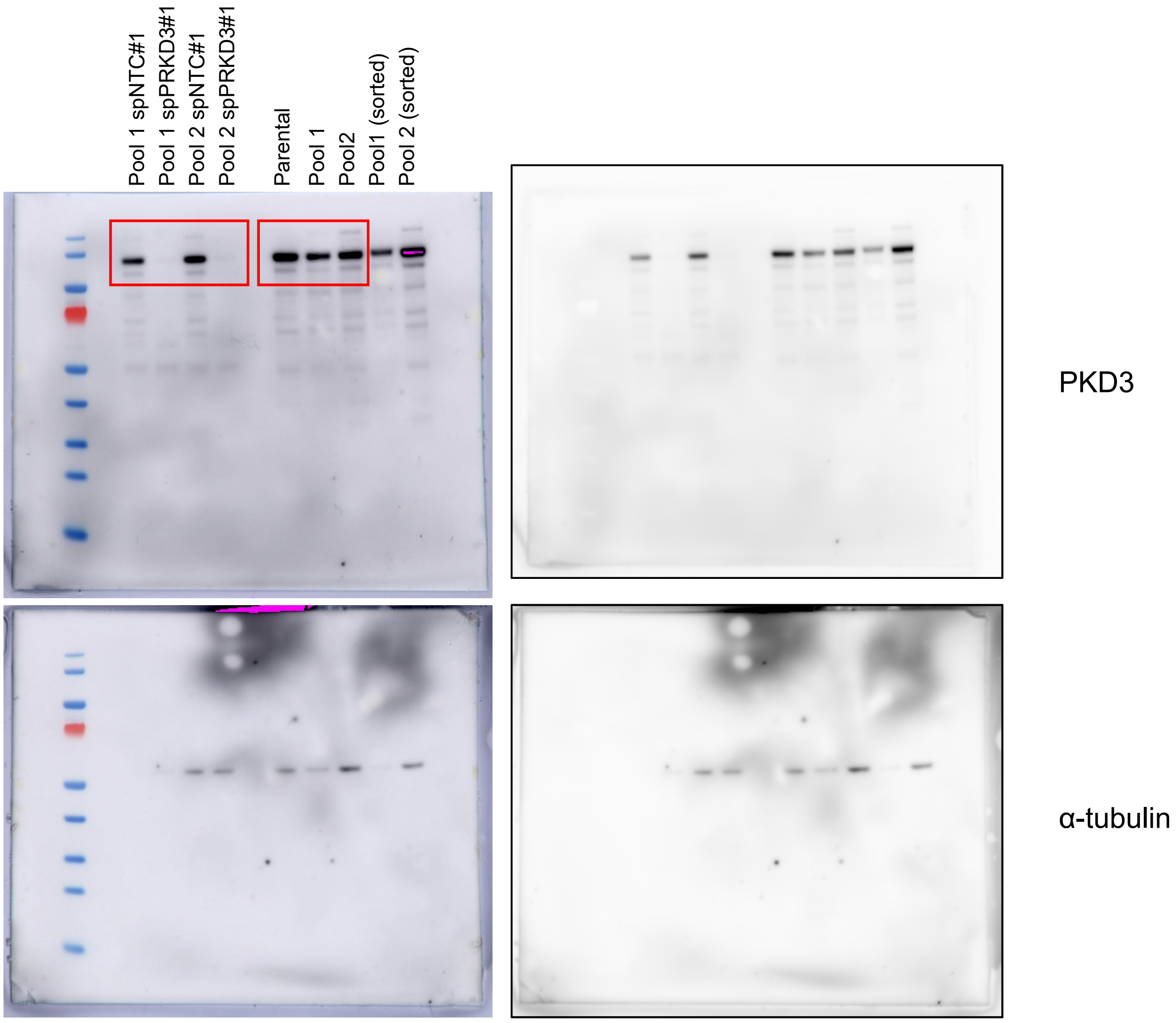

Unprocessed

Data Source – Figure S1E

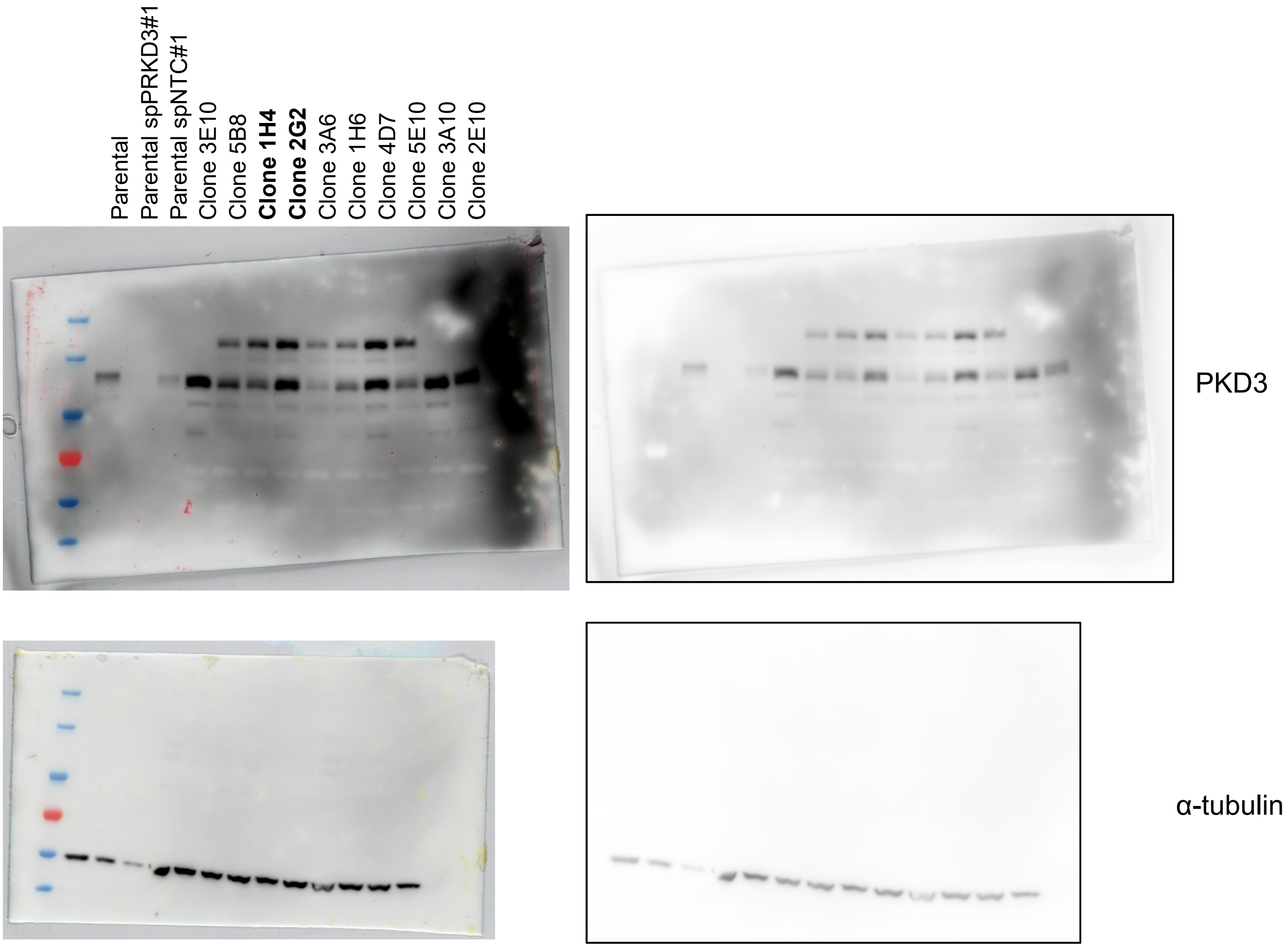

Unprocessed

Data Source – Figure 1F, Figure S1G

Unprocessed

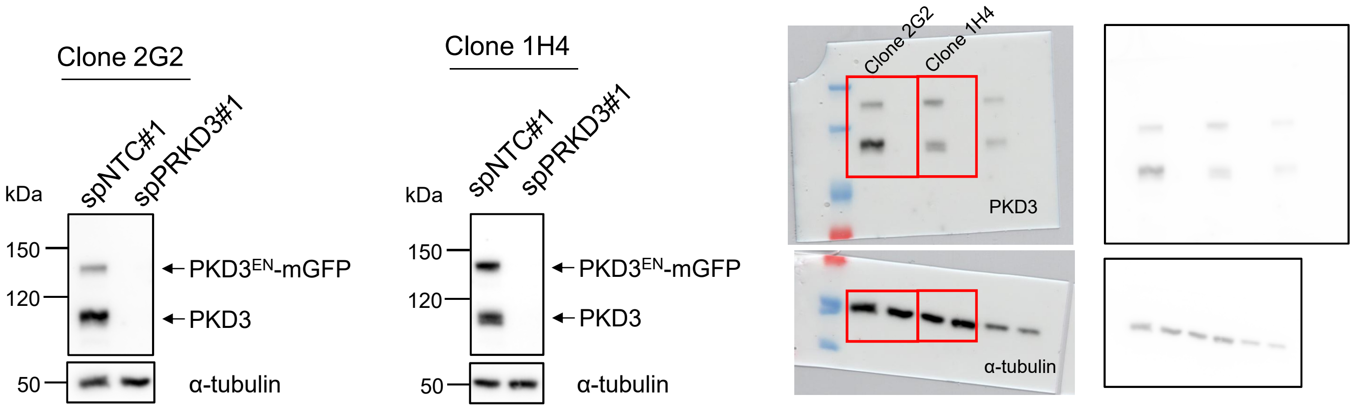

Data Source – Figure 2F

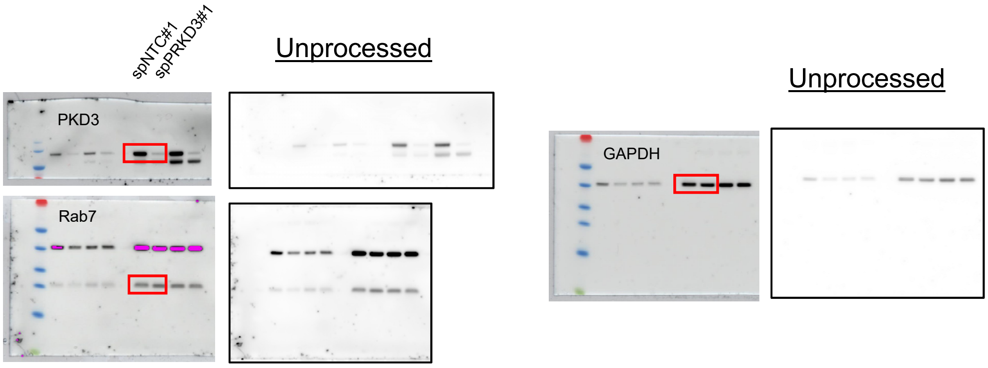

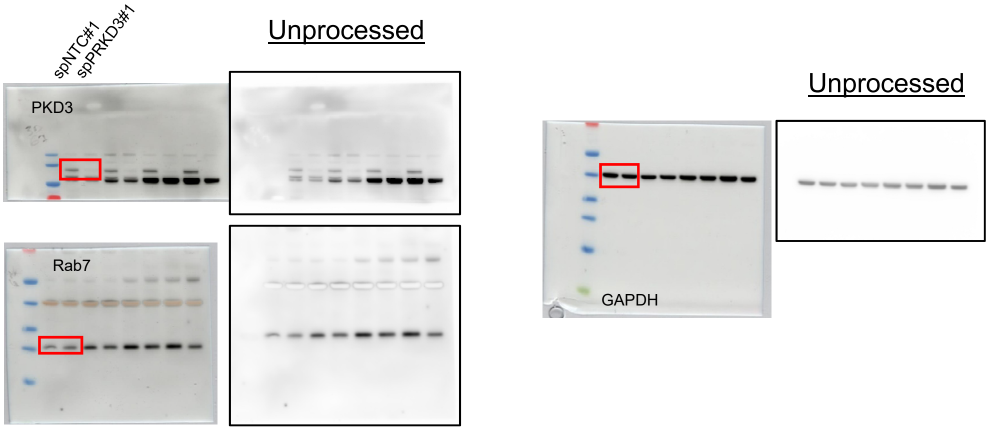

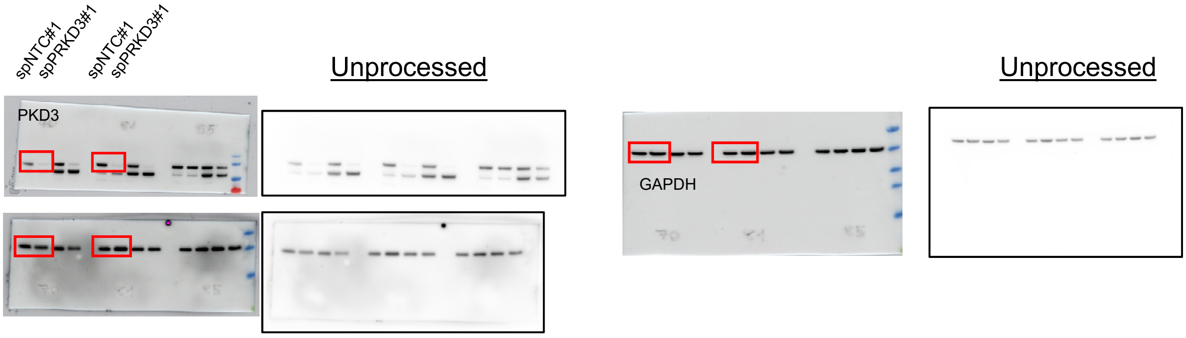

Data Source – Figure 2G

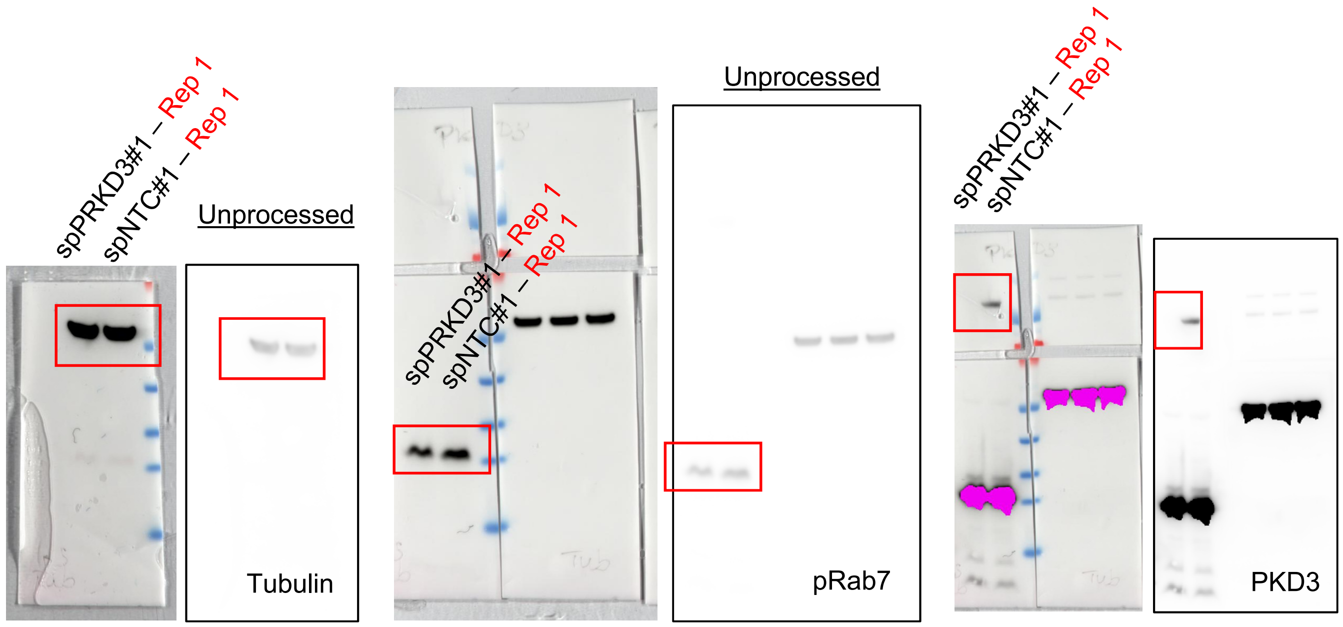

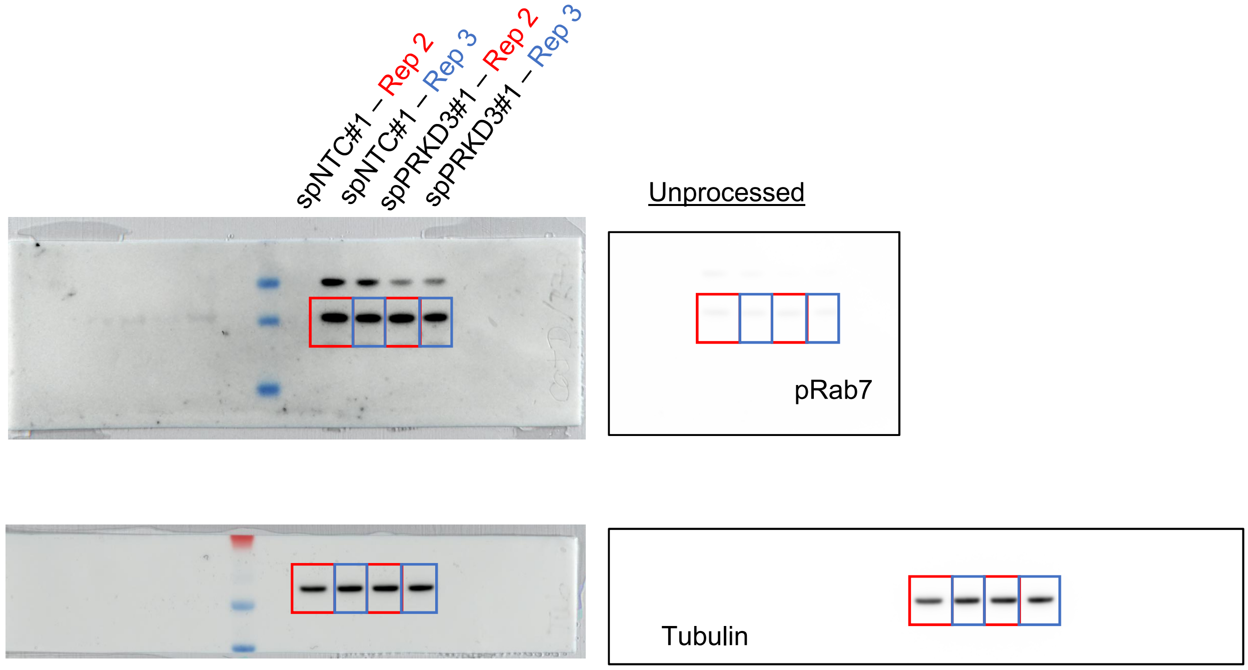

Data Source – Figure 2H

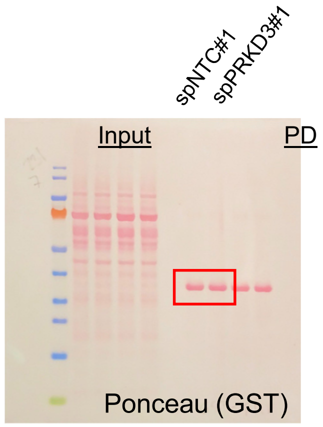

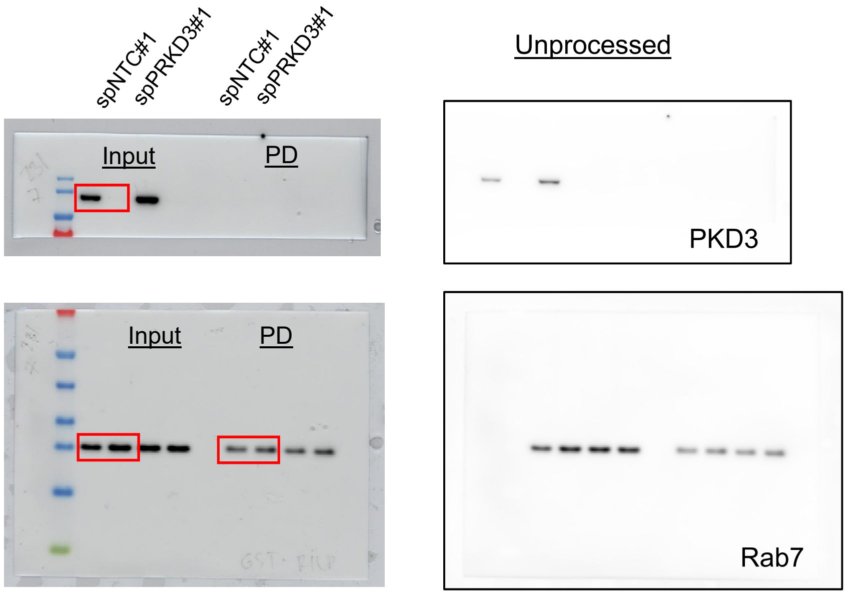

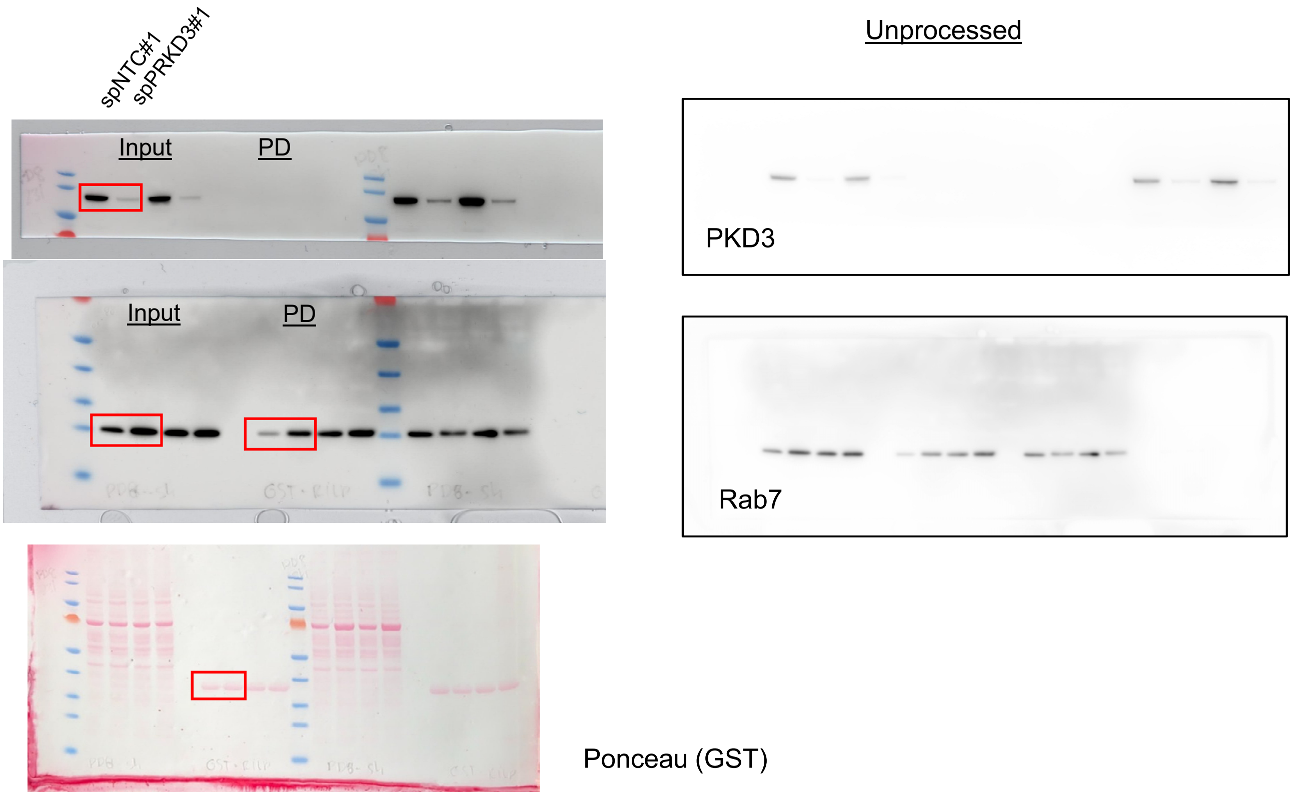

Data Source – Figure 2H

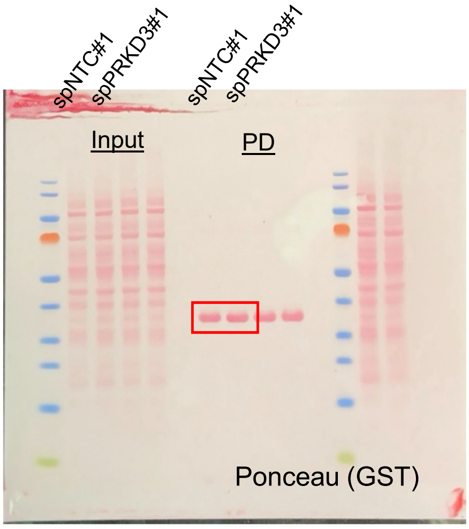

Unprocessed

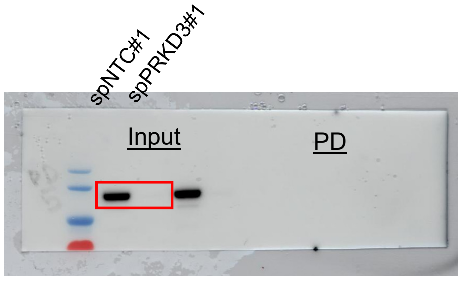

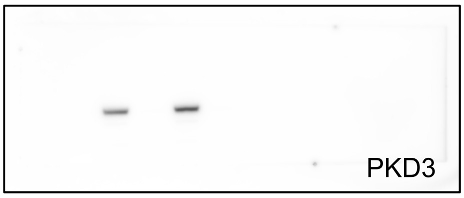

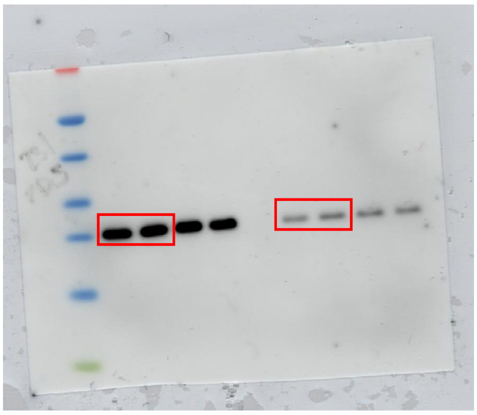

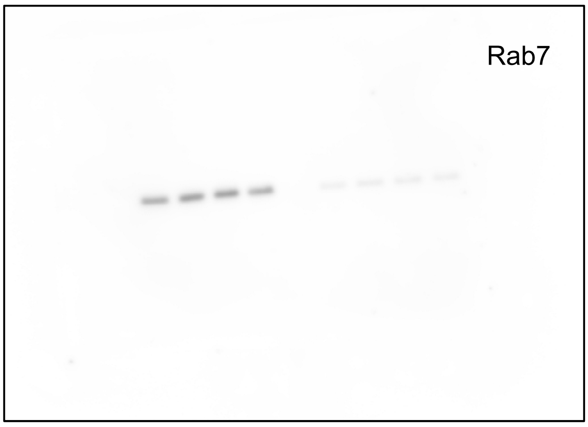

Data Source – Figure 3E

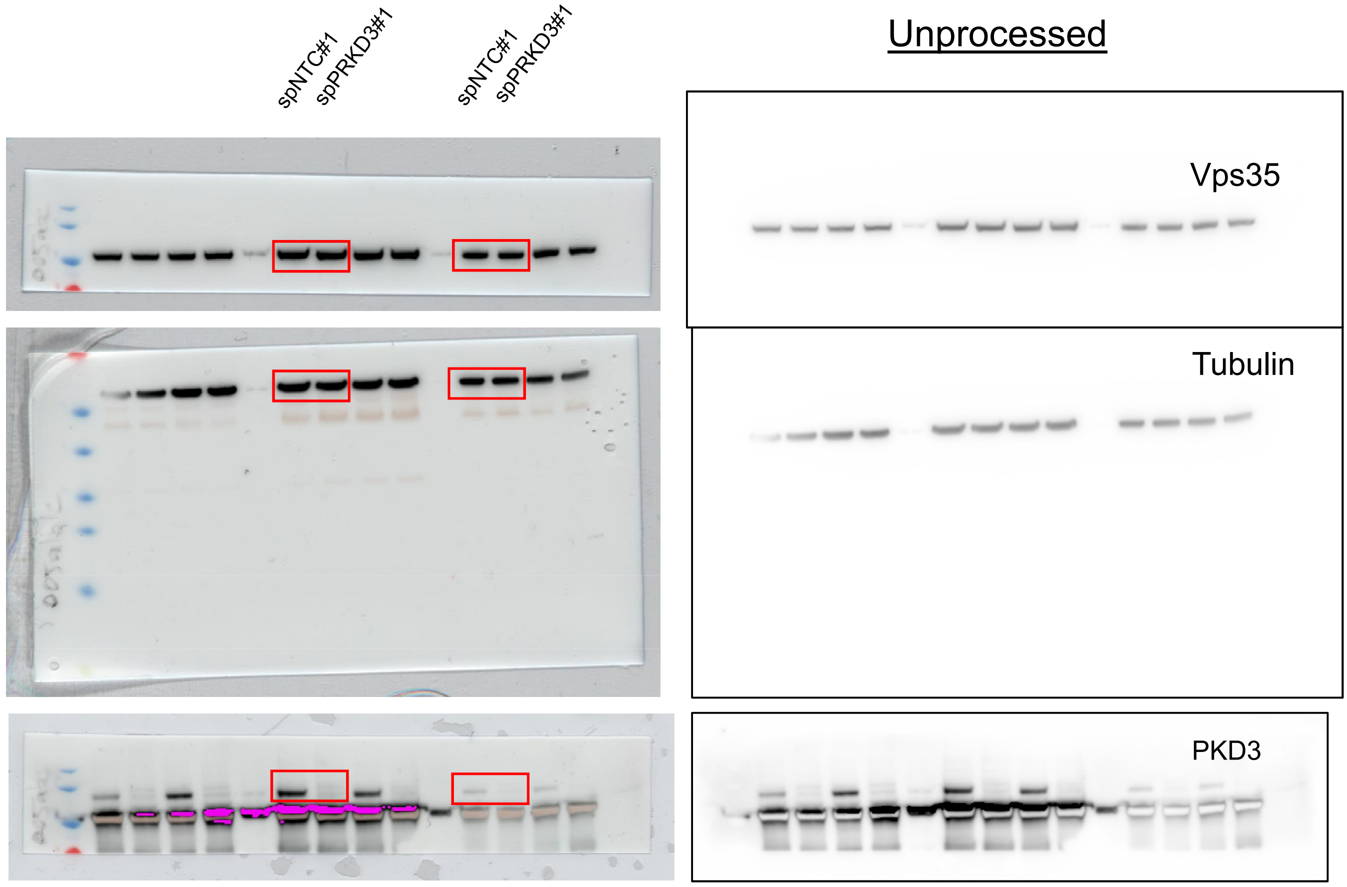

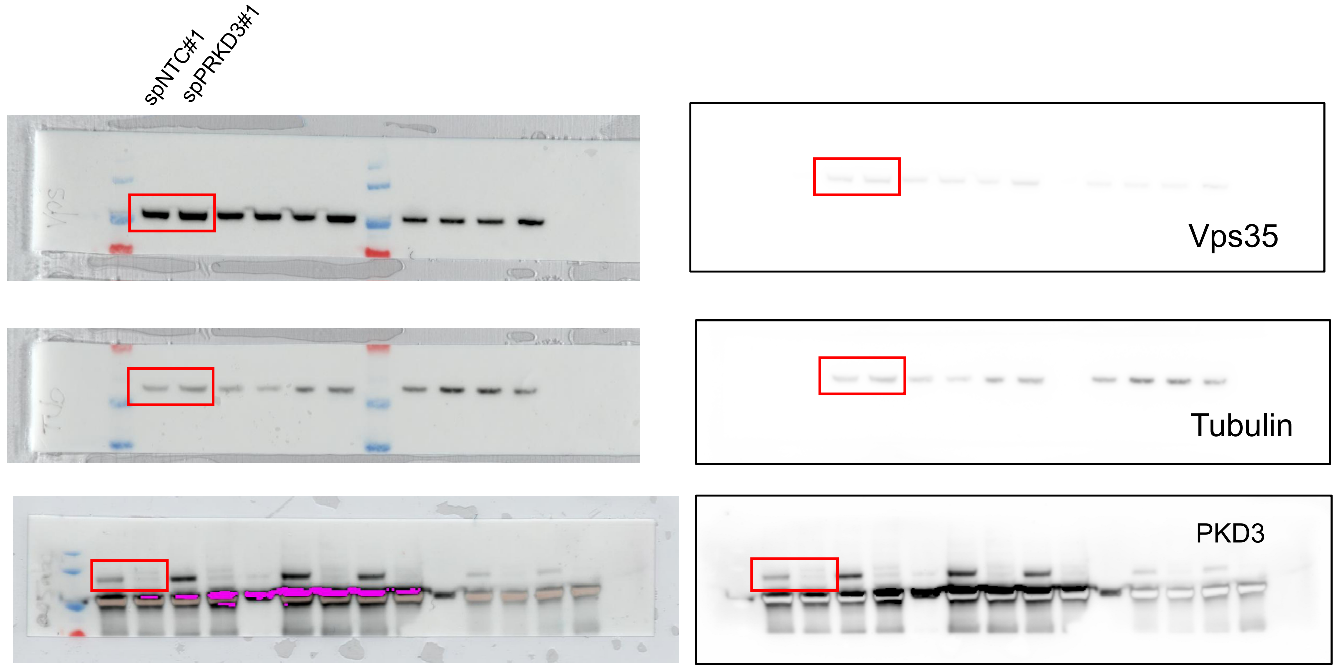

Data Source – Figure 3F

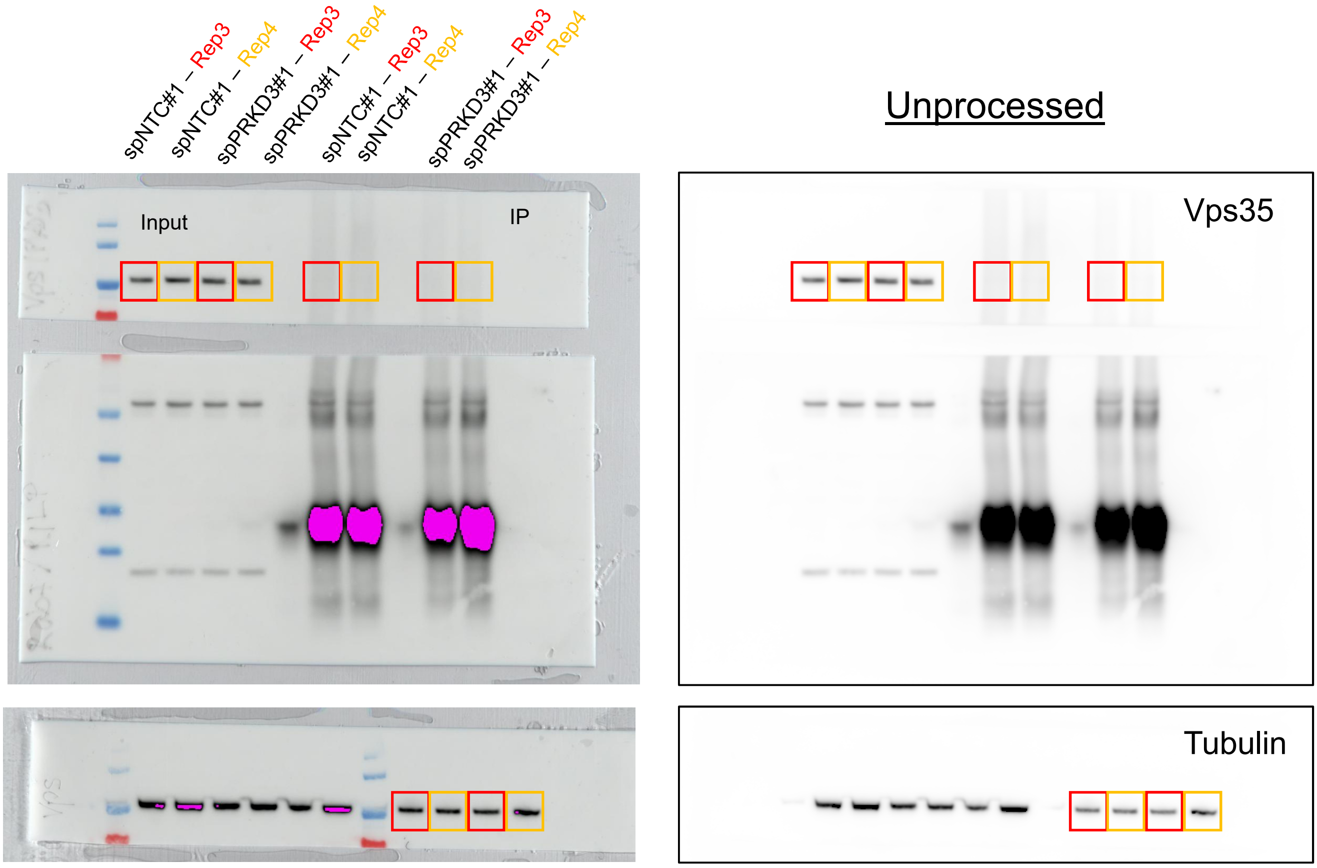

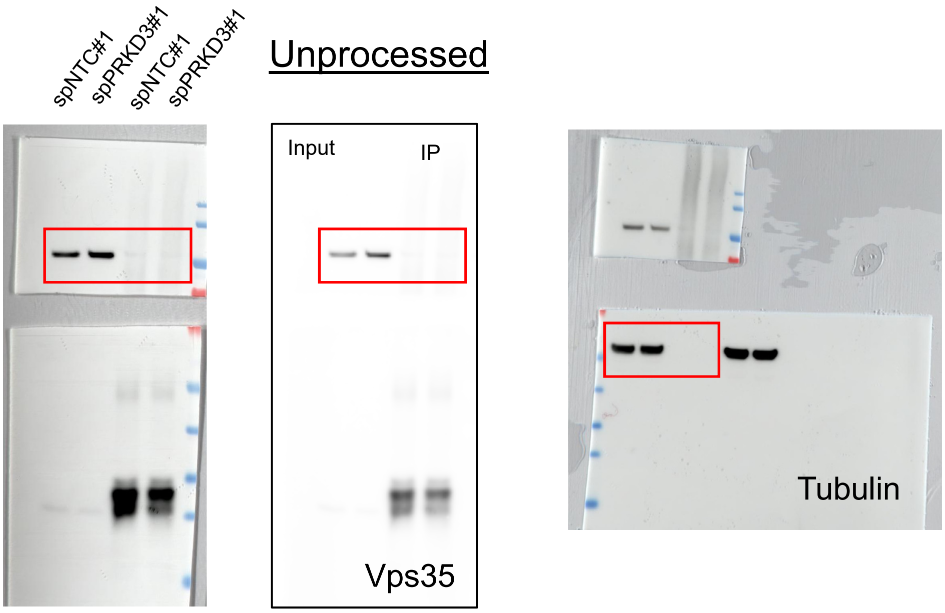

Data Source – Figure 3G

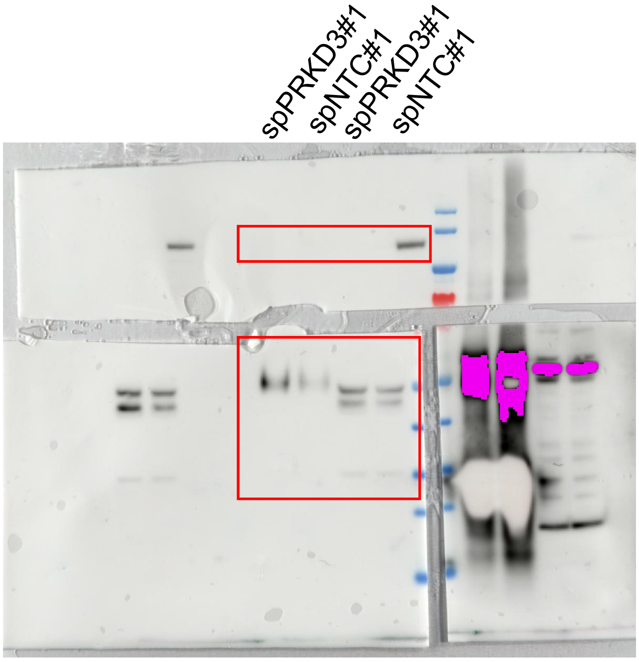

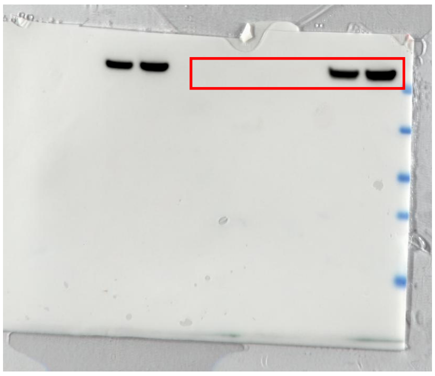

Unprocessed

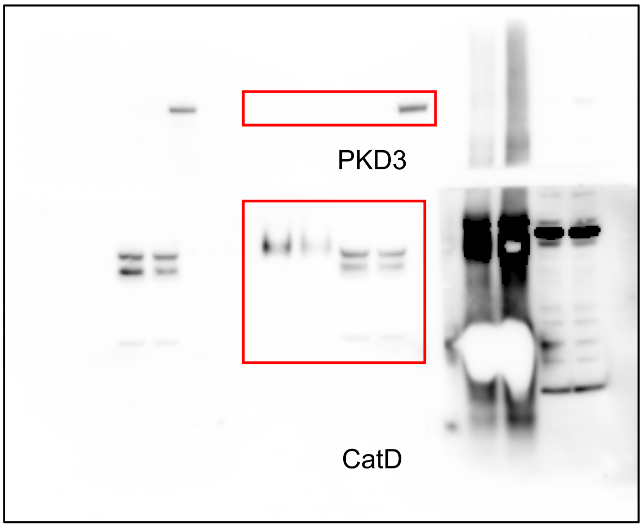

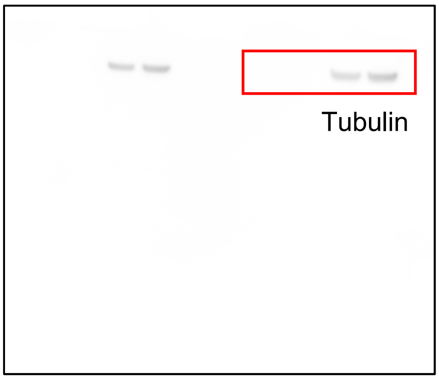

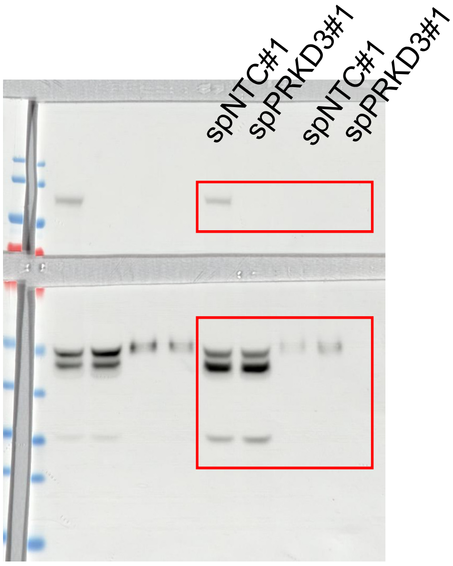

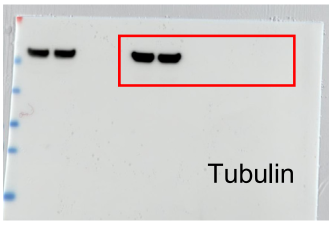

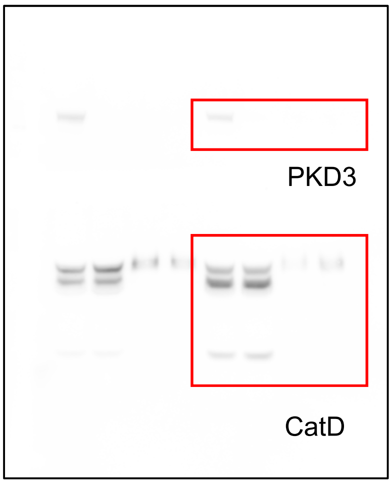

Data Source – Figure 3G

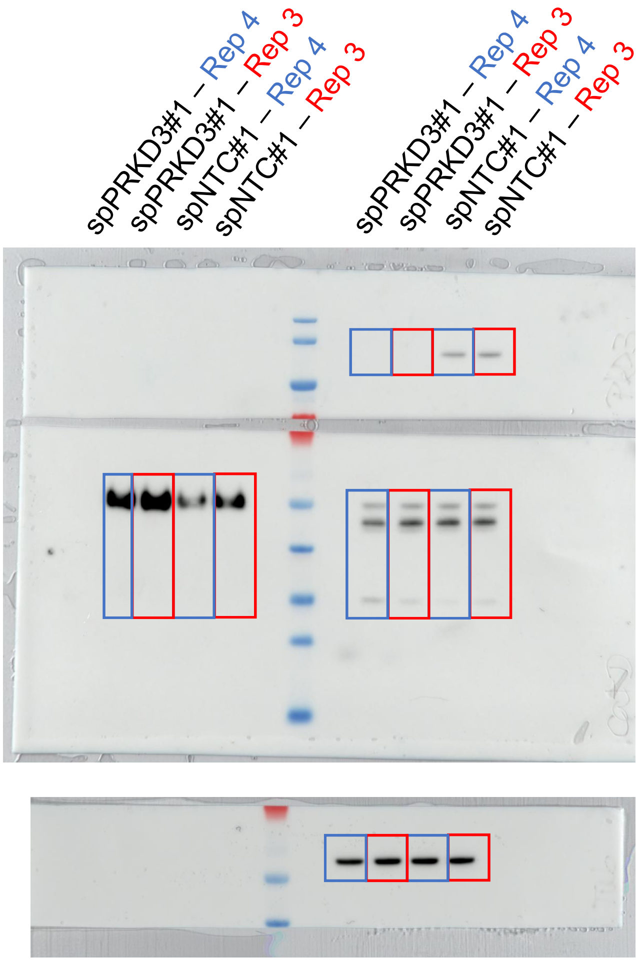

Unprocessed

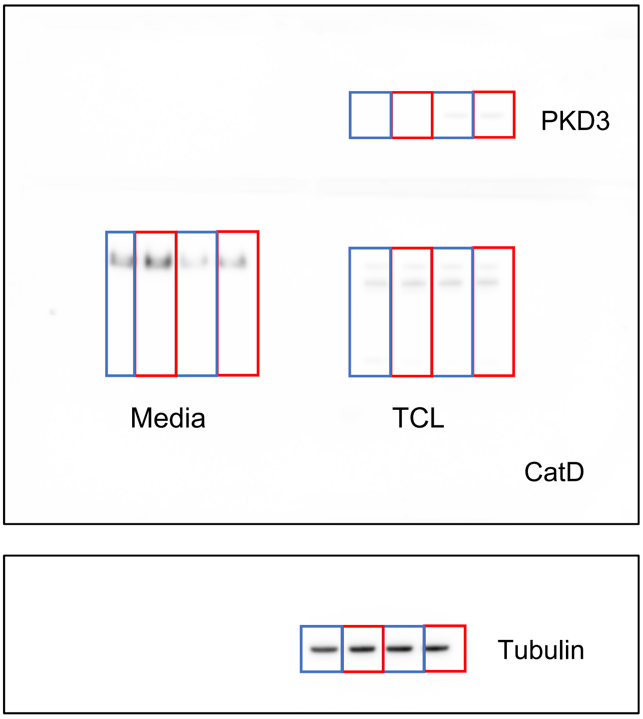

Data Source – Figure 4A

Unprocessed

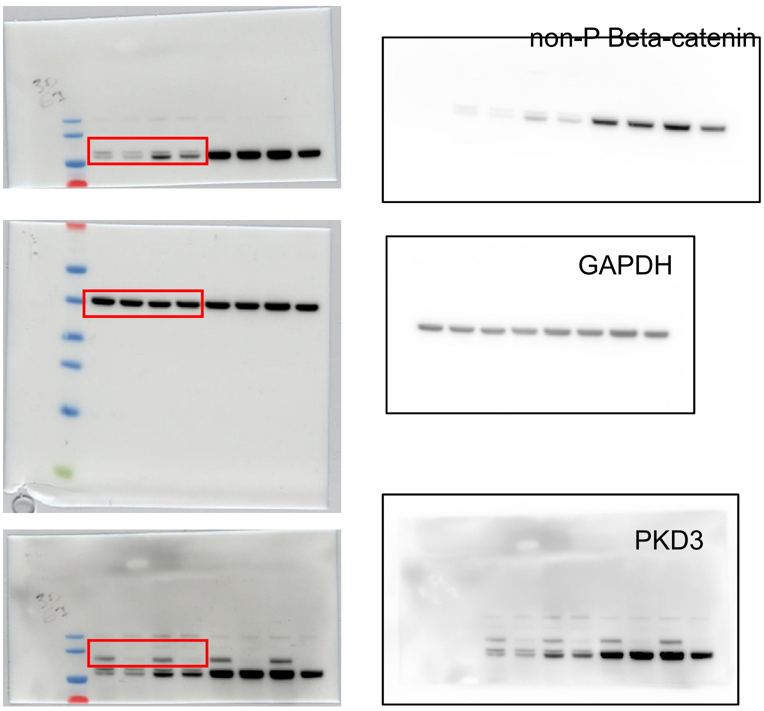

spNTC#1 spPRKD3#1 spNTC#1 spPRKD3#1 spNTC#1 spPRKD3#1 spNTC#1 spPRKD3#1

\- - + + - - + +

Unprocessed

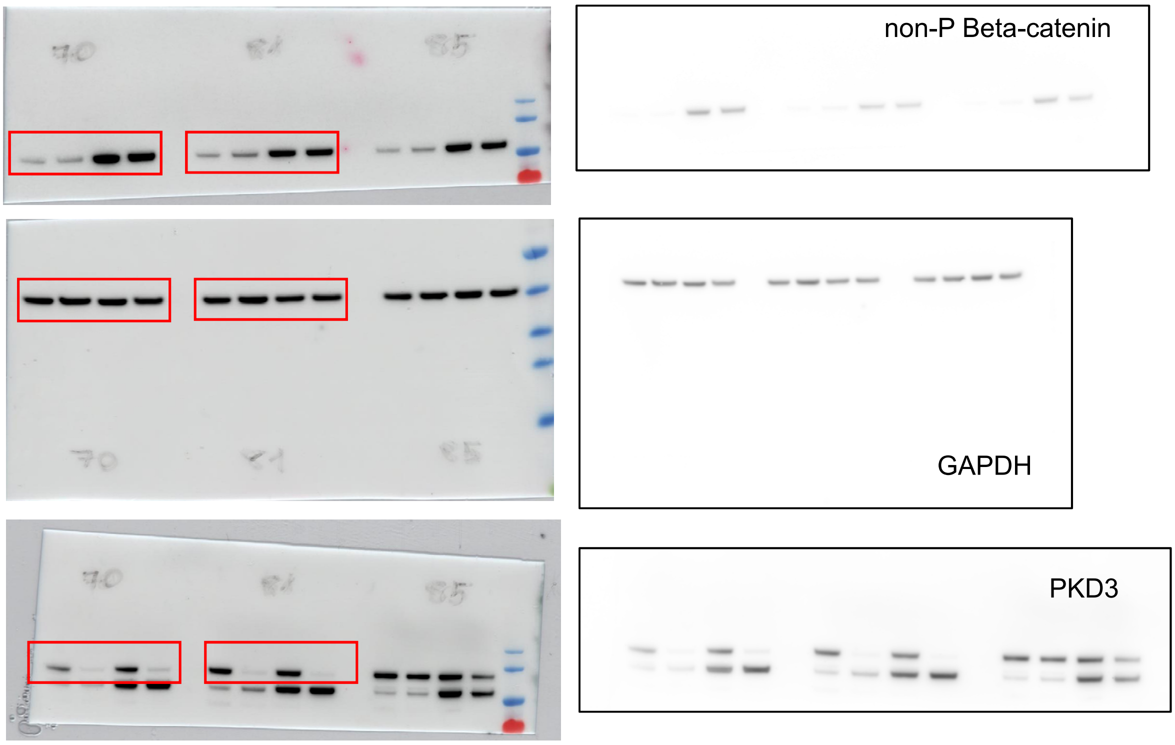

Data Source – Figure 4A

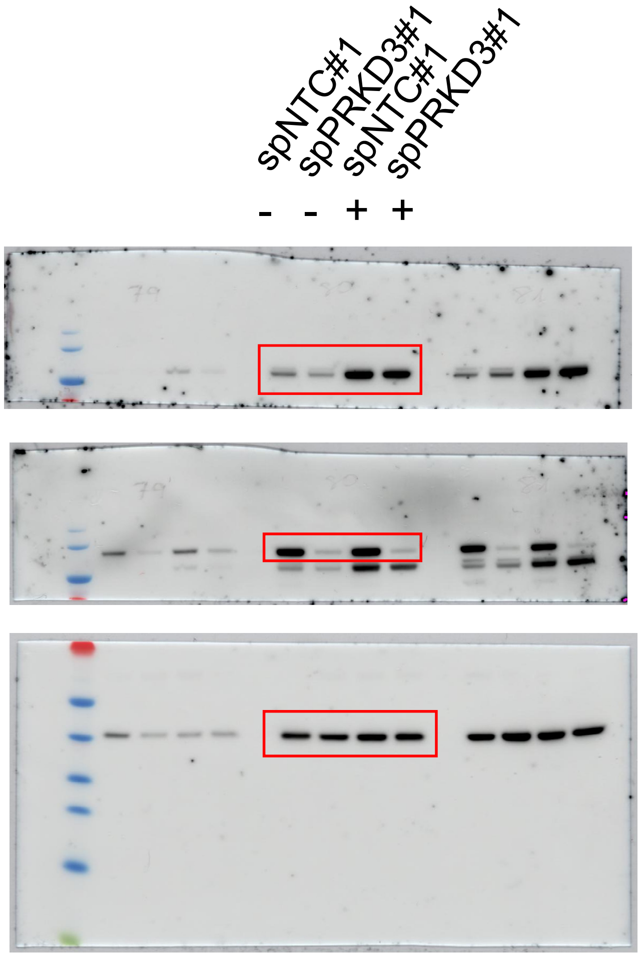

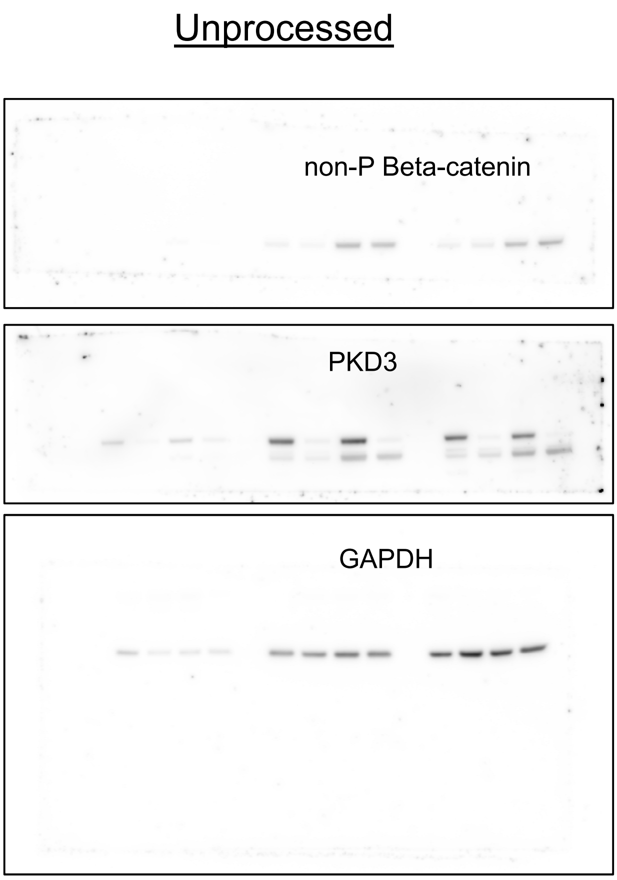

Data Source – Figure 4B

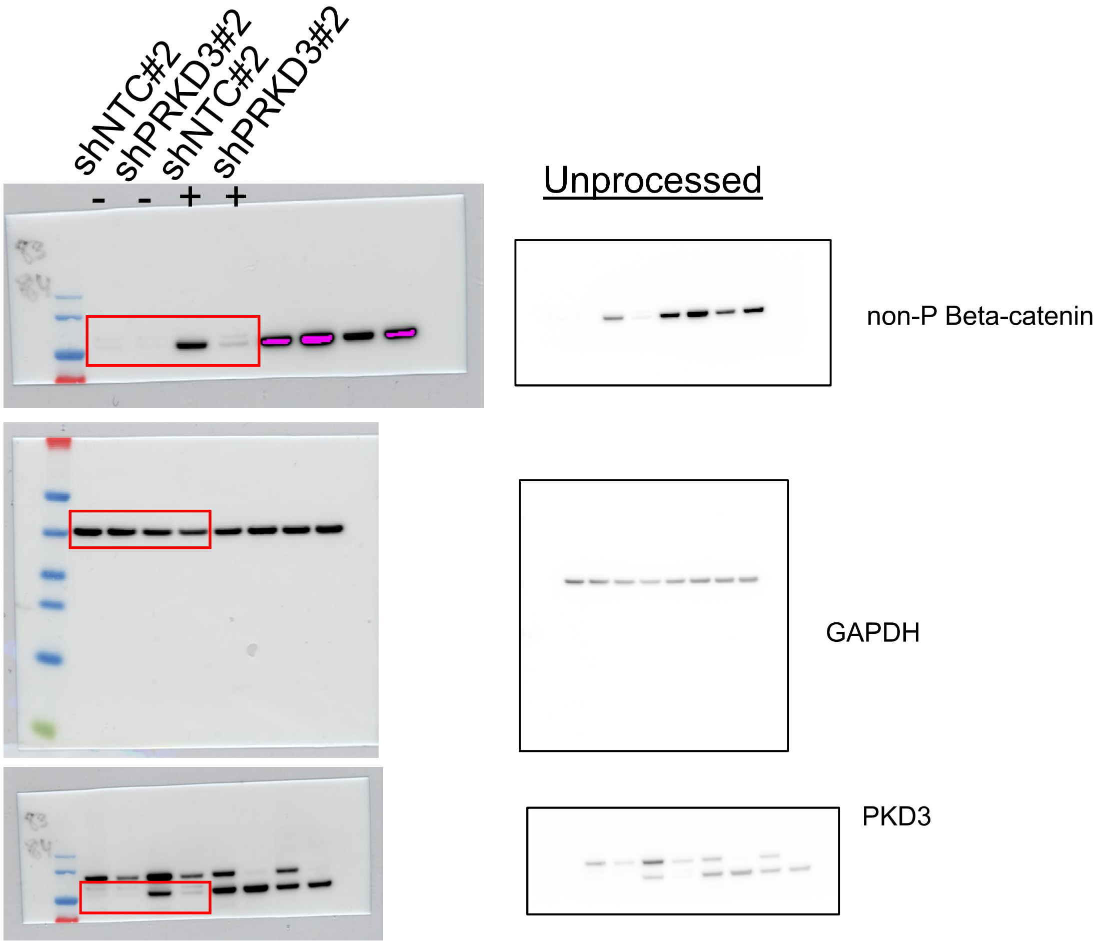

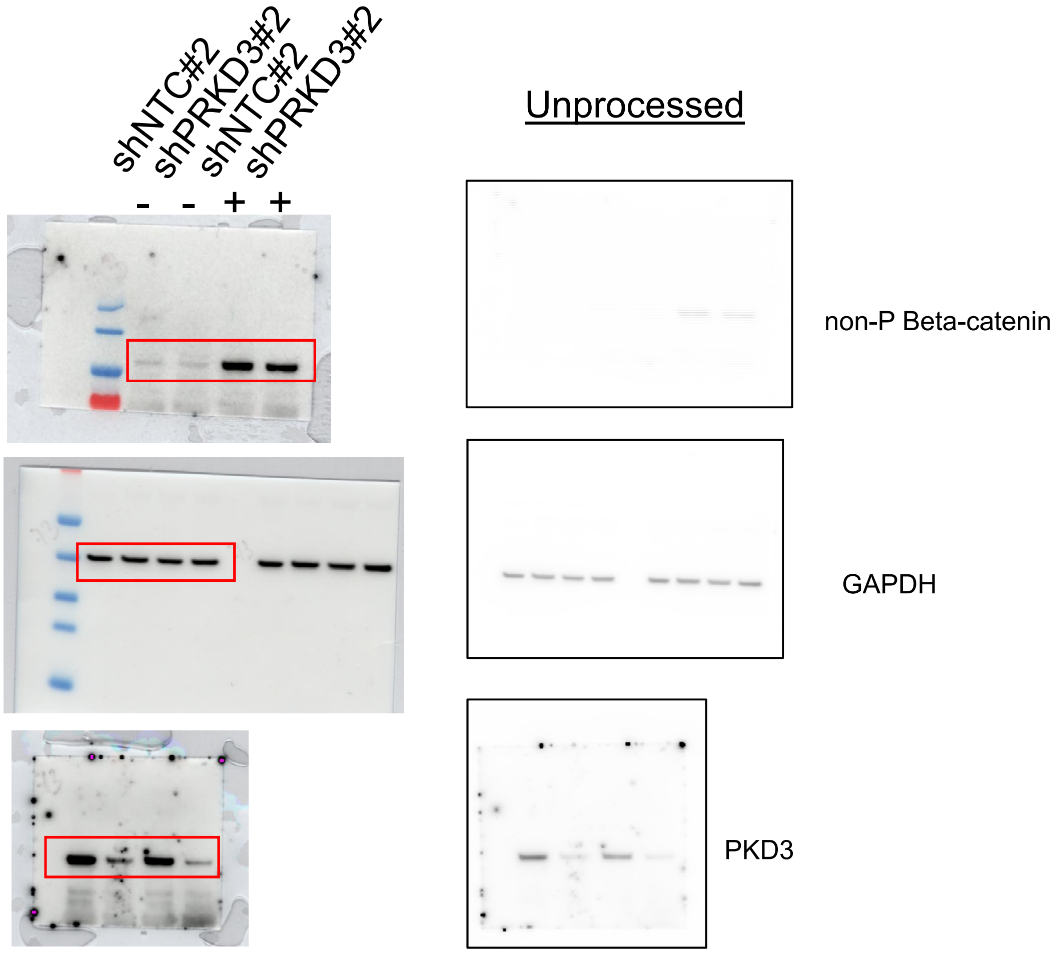

Data Source – Figure 4B

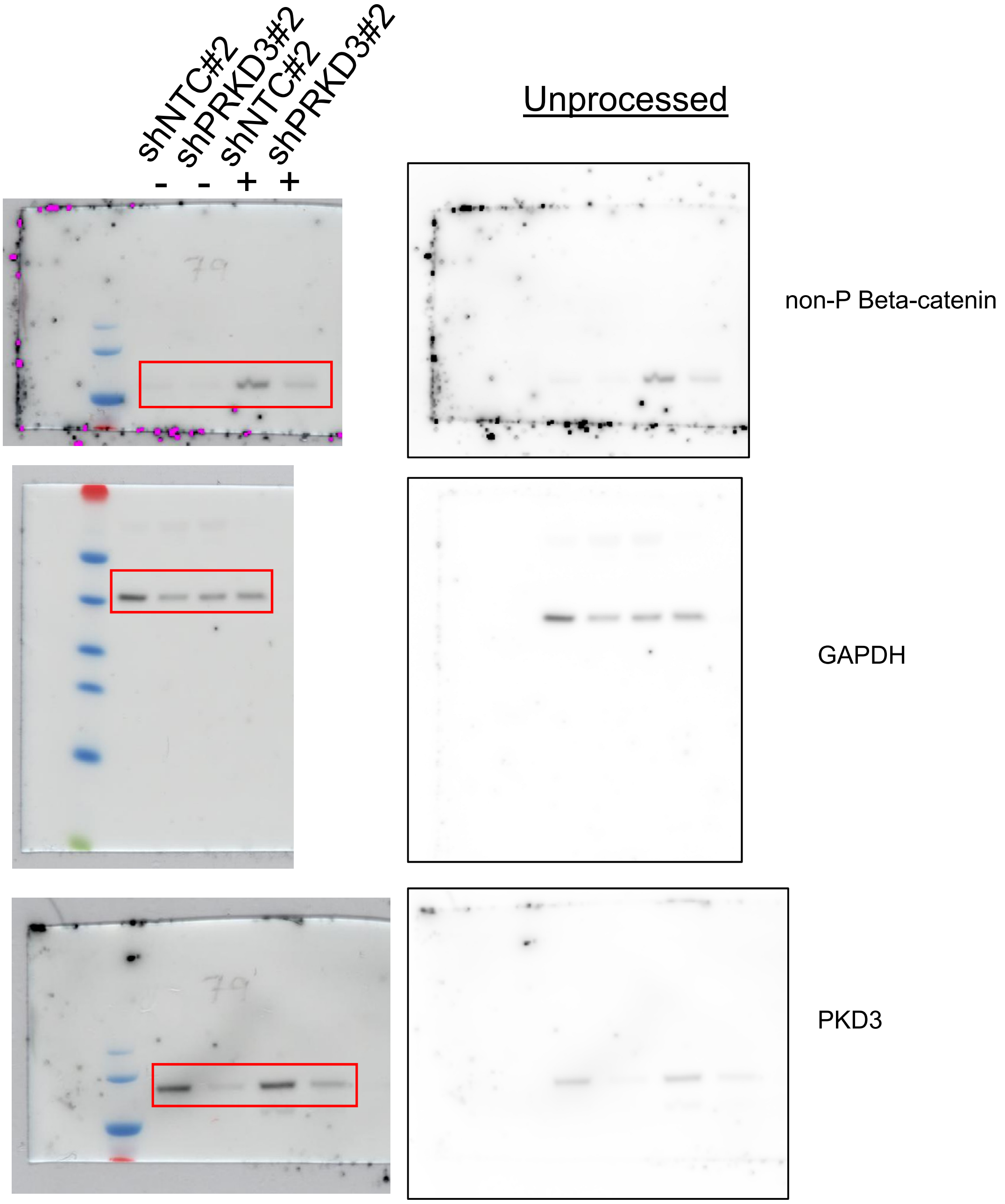

Data Source – Figure 4C

CRT - + - +
CHIR - - + +

<u>Unprocessed</u>

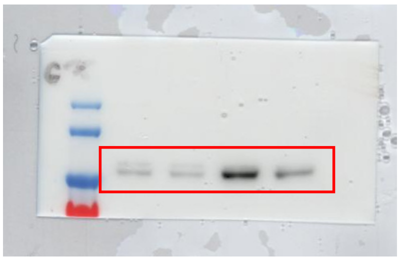

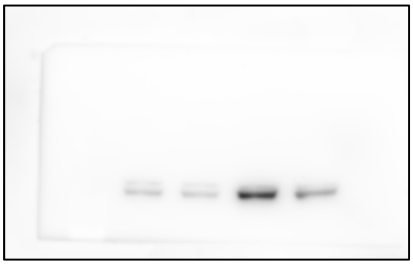

non-P Beta-catenin

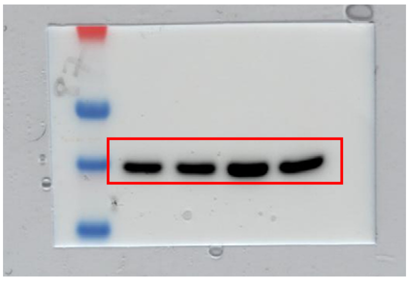

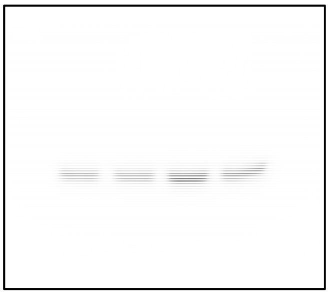

GAPDH

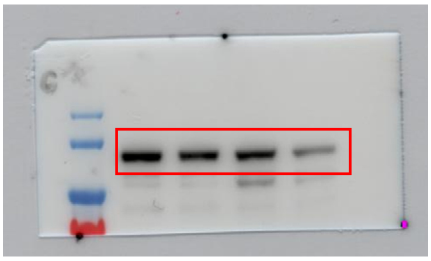

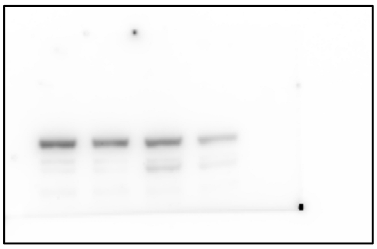

PKD3

CRT - + - +
CHIR - - + +

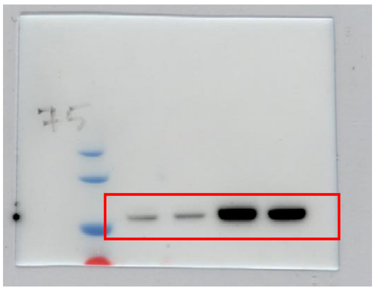

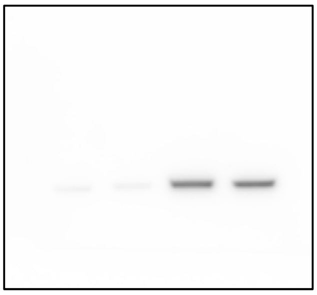

non-P Beta-catenin

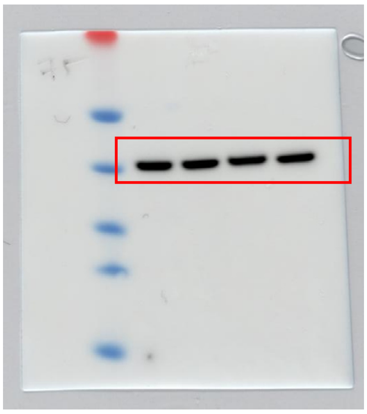

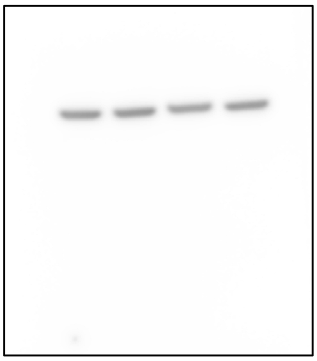

GAPDH

Data Source – Figure 4C

CRT	-	+	-	+
CHIR	-	-	+	+

Unprocessed

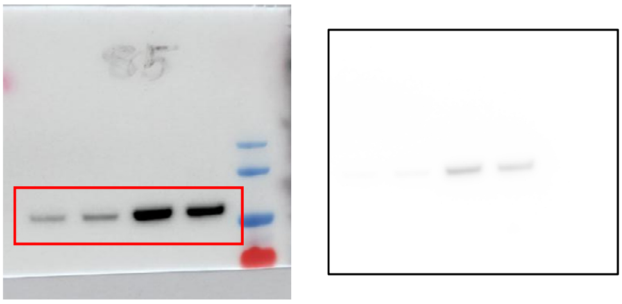

non-P Beta-catenin

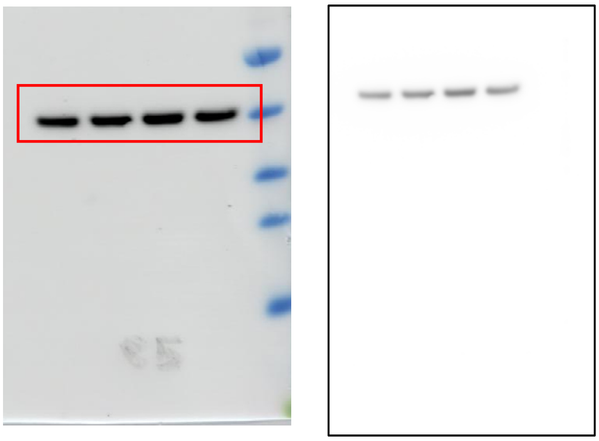

GAPDH

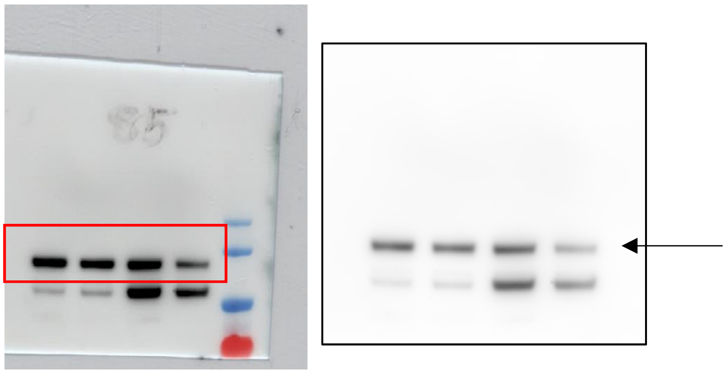

PKD3

Data Source – Figure S2D

spNTC#1 – Rep1
spPRKD3#1- Rep 1
spNTC#1 – Rep 2
spPRKD3#1 – Rep2
spNTC#1 – Rep3
spPRKD3#1 – Rep3
spNTC#1 – Rep4
spNTC#1 – Rep5
spPRKD3#1 – Rep4
spPRKD3#1 – Rep5

<u>Unprocessed</u>

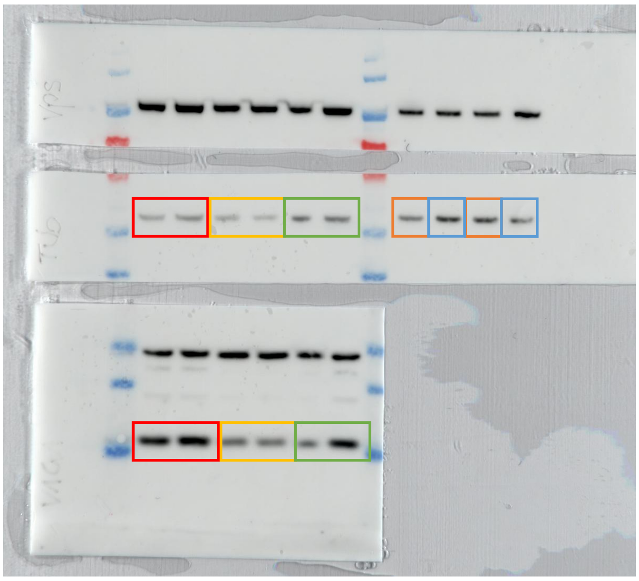

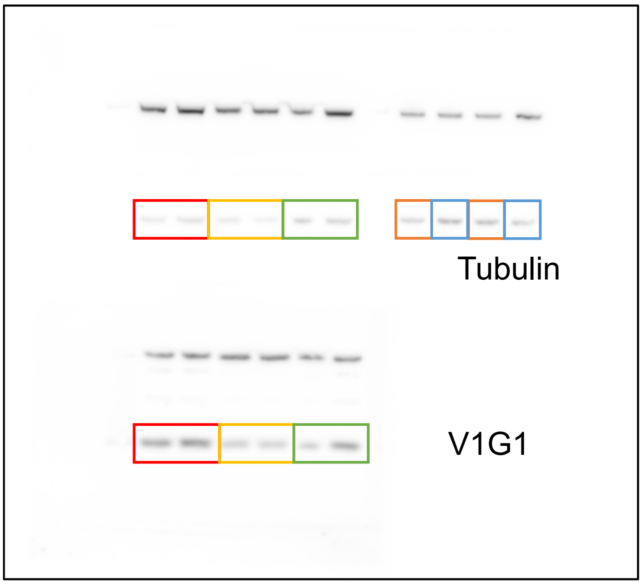

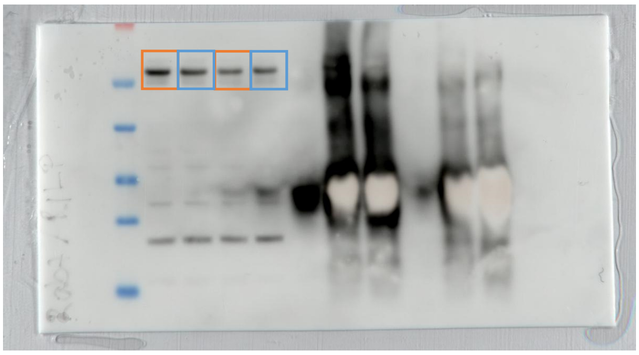

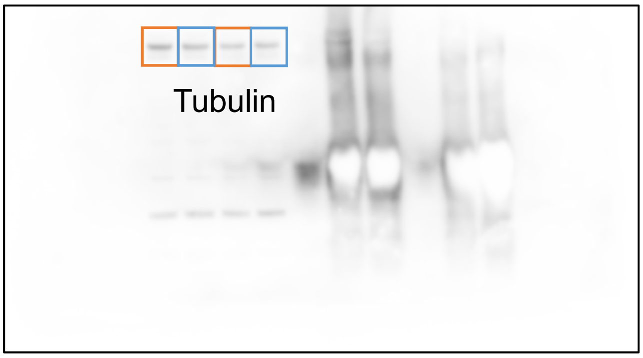

Supplement: Data S1. Uncropped Western blots, related to Figures 1, 2, 3, 4, S1, and S2 [file mmc2.zip › Data S1.pdf]
